# Diagnosis of Human Endemic Mycoses Caused by Thermally Dimorphic Fungi: From Classical to Molecular Methods

**DOI:** 10.3390/jof10090637

**Published:** 2024-09-06

**Authors:** Joaquina María García-Martín, Antonio Muro, Pedro Fernández-Soto

**Affiliations:** Infectious and Tropical Diseases Research Group (e-INTRO), Biomedical Research Institute of Salamanca-Research Centre for Tropical Diseases at the University of Salamanca (IBSAL-CIETUS), Faculty of Pharmacy, University of Salamanca, 37007 Salamanca, Spain; ama@usal.es (A.M.); pfsoto@usal.es (P.F.-S.)

**Keywords:** diagnostic methods, infectious fungal diseases, blastomycosis, coccidioidomycosis, emergomycosis, histoplasmosis, paracoccidioidomycosis, talaromycosis, sporotrichosis

## Abstract

Human endemic mycoses are potentially fatal diseases caused by a diverse group of fungi that can alter their morphology in response to an increase in temperature. These thermally dimorphic fungi affect both healthy and immunocompromised hosts, causing a substantial health and economic burden. Despite this, the diagnosis of endemic mycoses is still a formidable challenge for several reasons, including similar symptomatology, limited utility of classical diagnostic methods, inaccessibility to reliable molecular approaches in most endemic areas, and a lack of clinical suspicion out of these regions. This review summarizes essential knowledge on thermally dimorphic fungi and the life-threatening diseases they cause. The principle, advantages and limitations of the methods traditionally used for their diagnosis are also described, along with the application status and future directions for the development of alternative diagnostic strategies, which could help to reduce the disease burden in endemic areas.

## 1. Introduction

**Figure 1:**
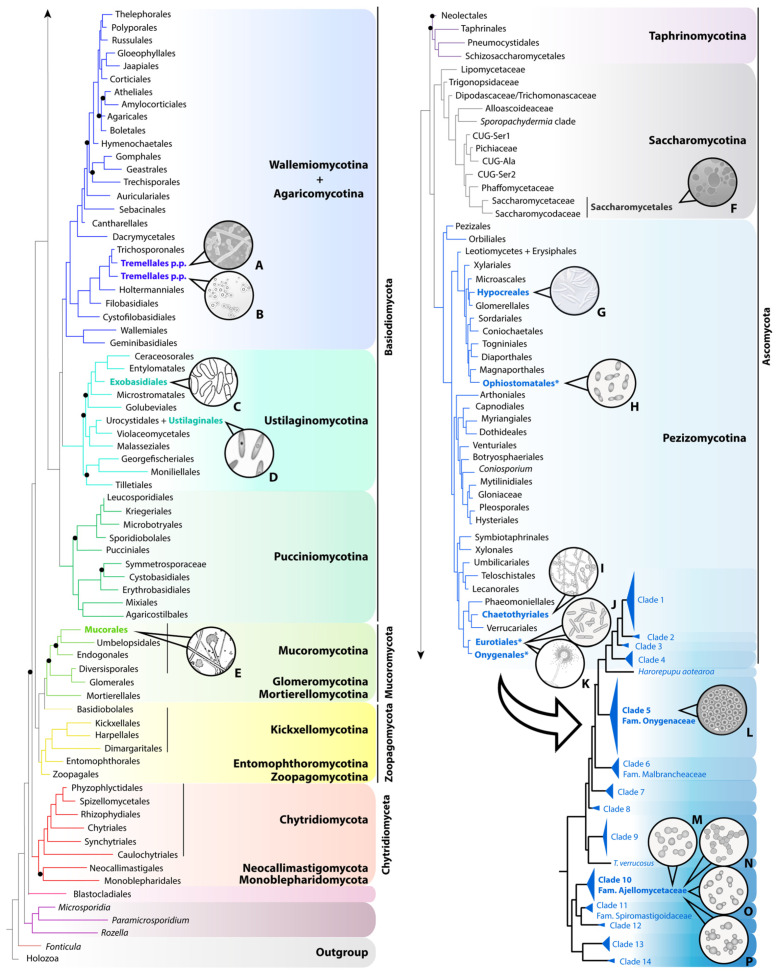
Distribution of relevant human pathogenic species across fungi, based on the phylogenomic tree published by Li and colleagues [4] and the multigene phylogeny of the order Onygenales published by Kandemir and collaborators [5]. In the genome-scale tree of fungi, derived from a concatenated data matrix (290 genes), the nine lineages currently accepted are named and shaded in different colors. Terminals of the tree are labeled using order-level taxonomic names, except for Saccharomycotina, for which informal and family-level names of the major clades are used. Orders including pathogenic species appear in bold and are colored, with those three comprising thermally dimorphic fungi of clinical interest marked with an asterisk. The phylogeny of Onygenales (lower right corner of the figure) was obtained by combining eight loci. Only those clades comprising human pathogens are formally named. A. *Trichosporon* spp. B. *Cryptococcus* spp. C. *Exobasidium* spp. D. *Ustilago* spp. E. *Mucor* spp. F. *Candida* spp. G. *Fusarium* spp. H. *Sporothrix* spp. I. *Fonsecaea* spp. J. *Talaromyces marneffei*. K. *Aspergillus* spp. L. *Coccidioides* spp. M. *Blastomyces* spp. N. *Emergomyces* spp. O. *Histoplasma* spp. P. *Paracoccidioides* spp.

Since the emergence of fungi ca. 700 million years ago, pathogenic forms have emerged independently in multiple lineages during evolution. Among them, there is a taxonomically diverse group of fungi distributed across four of the nine fungal lineages currently accepted ([1]; Figure 1) that drastically alter their morphology and developmental programs in response to different environmental stimuli, such as the concentration of nutrients, ammonium, O_2_ and CO_2_, pheromones, pH, or temperature (Table S1). Because of this morphological transition, which entails changes in the cell wall composition to ensure fungal survival [2], they are known as dimorphic fungi.

Given their impacts on public health, this review focuses on thermally dimorphic fungi (hereafter TDF), i.e., species that switch between two morphologies depending on the temperature at which they grow, and presents a summary of the methods currently available for the diagnosis of their associated diseases. Specifically, the TDF included here are primary pathogens of the genera *Blastomyces*, *Coccidioides*, *Histoplasma*, *Paracoccidioides*, *Sporothrix*, *Talaromyces*, which need to be handled in biosafety level 3 laboratories (BSL-3), and opportunistic species of *Emergomyces* (BSL-2), a recently described genus [3]. These fungi are responsible for different human systemic mycoses that can present as localized or disseminated and are associated with a variety of symptoms, including severe lung damage and stigmatizing cutaneous lesions (Table 1). Note that some close relatives to the species included in Table 1, such as *Blastomyces silverae* or *Paracoccidioides ceti*, cause pulmonary infections in animals, but have been only occasionally reported as human pathogens, and therefore will not be treated here.

Most TDF belong to the family Ajellomycetaceae, a unique clade within the monophyletic order Onygenales, as it includes the highest number of species adapted to survival and replication within mammalian hosts [11,34]. The exceptions are *Coccidioides* (family Onygenaceae, Onygenales), *Sporothrix* (order Ophiostomatales) and *Talaromyces marneffei* (order Eurotiales). TDF generally develop as spore-producing mycelia (saprophytic phase) in nature (~25–30 °C) or when incubated at similar temperatures, and transform into yeast-like cells (parasitic phase) when infecting a host. In the case of *Coccidioides*, a special type of spore called arthroconidia, produced by segmentation of hyphae, convert into large parasitic structures (spherules) comprising numerous endospores that are cyclically released within the host [35]. Meanwhile, *Emergomyces* is characterized by a spectrum of infective forms from budding yeasts to adiaspores, i.e., large, thick-walled non-replicating structures resulting from the growth of inhaled spores that lead to the formation of granulomas [17].

Regardless the type of structure to which spores transform, morphological conversion is essential for the upregulation of genes involved in subverting host immune defenses and in the increased expression of virulence factors (Table S2). These gene products are not necessary for the growth of the parasitic phase in vitro, but are essential for fungal survival within the host [36], as they enable immune response evasion and host colonization.

The outcome of the infections caused by TDF not only depends on the immune status of the host but also on the dose of infectious particles. For this reason, unlike most fungal pathogens, TDF can affect both immunocompromised and healthy individuals if enough spores are inhaled. Moreover, TDF may persist as latent infections for years and reactivate when the immune system weakens (due to ageing, concomitant diseases, or immunosuppressive treatments).

**Figure 2:**
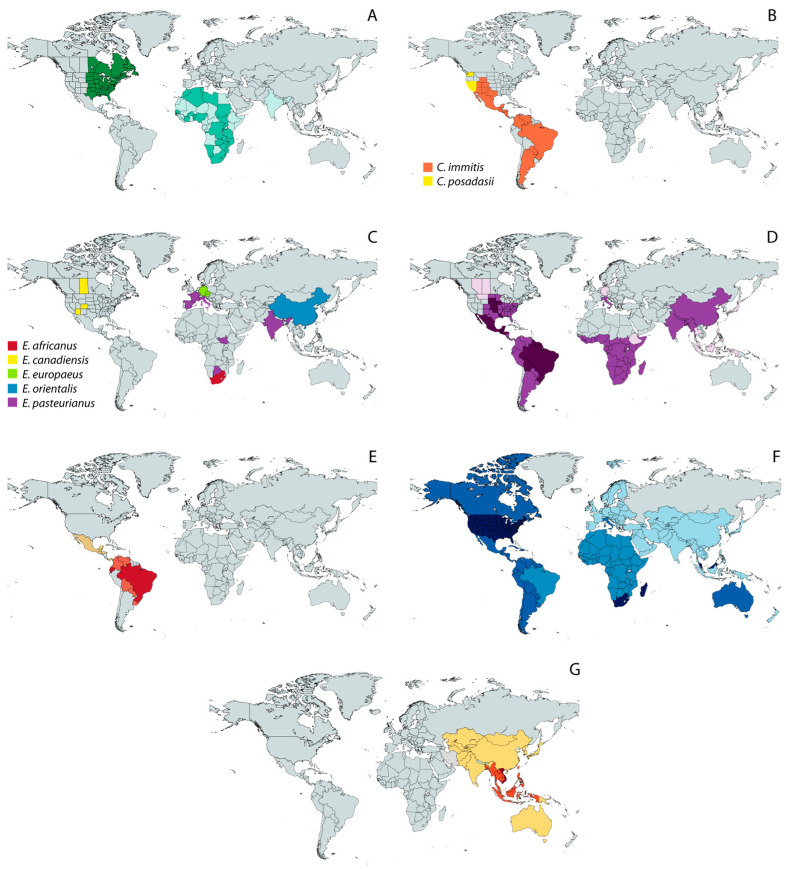
Geographic distribution of endemic mycoses based on published data [18,39,51,52,53,54]. (**A**) Blastomycosis; (**B**) Coccidioidomycosis; (**C**) Emergomycosis; (**D**) Histoplasmosis; (**E**) Paracoccidioidomycosis; (**F**) Sporotrichosis; and (**G**) Talaromycosis. Except for the maps of coccidioidomycosis (**B**) and emergomycosis (**C**), in which different colors represent different species, color shades represent different levels of incidence, with darker tones corresponding to the highest incidence.

Historically, TDF have been considered regionally endemic, that is, they have been thought to occur in limited geographic ranges (Figure 2), being responsible for substantial morbidity and mortality in their respective regions, i.e., tropical areas of Africa, Asia, and Central and South America [37]. As such, the infections caused by TDF are commonly referred to as “endemic mycoses”. However, due to climate change, the spatial range of different TDF is widening [38,39], which contributes to the already difficult task of diagnosing TDF-caused infections because physicians may have not been in contact with these etiological agents before.

The diagnosis of endemic mycoses is challenging per se due to considerable clinical overlap among them. Indeed, symptoms are not only nonspecific but also hardly distinguishable from those associated with unrelated diseases. For instance, some endemic mycoses, including blastomycosis, coccidioidomycosis and histoplasmosis, are frequently misdiagnosed as pulmonary or intestinal tuberculosis [17,40,41], bacterial or viral pneumonia [42,43], bowel disease [44,45], or diverse malignancies [6,46,47]. In turn, sporotrichosis often presents as lymphocutaneous lesions mimicking those caused by atypical mycobacterial infections, nocardiosis, and leishmaniasis [30].

Classical diagnostic methods are not as accurate as desired, which, coupled with the mentioned syndromic similarities, makes a high index of suspicion crucial to ensure pathogen identification and appropriate treatment [12], avoiding unnecessary antimicrobial use and development of antibiotic resistance. This is especially true in non-endemic areas, where cases associated with immigrants and travelers who returned from endemic countries [48,49] may be undetected or misdiagnosed due to a lack of awareness. On the other hand, reliable molecular diagnostic methods are not available in most endemic areas.

Furthermore, given that person-to-person transmission has not been documented, except for rare cases of transmission by organ transplantation [50], endemic mycoses are not notifiable diseases.

Notably, zoonotic transmission of sporotrichosis through bites or scratches from infected cats is now considered a public health problem in hyperendemic areas [50,55], which makes a prompt and accurate diagnosis necessary to break the chain of transmission and to improve epidemiological knowledge. In this regard, the disease burden and distribution of *Sporothrix* and other TDF, specially the recently described genus *Emergomyces*, are probably much larger than currently estimated [19]. In the USA alone, TDF of the order Onygenales collectively cause more than 650,000 new infections each year [8], and the global disease burden continues to rise yearly [56], even if numerous cases remain underdiagnosed and neglected in some endemic regions [57,58].

As for the treatment of TDF-related mycoses, four main classes of antifungal drugs are currently available: (1) polyenes, such as amphotericin B (AmB), available in different intravenous formulations like liposomal (L-AmB) or deoxycholate (AmB-d), which inhibit fungal growth by forming pores in the membranes that lead to cytoplasm leakage; (2) azoles, such as fluconazole (FLZ), itraconazole (ITZ), voriconazole (VRC), or posaconazole (PSZ), which interfere with the biosynthesis of ergosterol, a key cell membrane component; (3) echinocandins, including caspofungin, micafungin, and anidulafungin, are less toxic and inhibit cell wall synthesis, forcing cell rupture and/or aberrant hyphal growth; and (4) synthetic antimetabolites, like flucytosine, which inhibit DNA and RNA synthesis, thus causing cell death [59]. Additionally, several new drugs are in clinical trials [17,60].

In general, antifungal therapy is effective, with AmB and ITZ being the most frequently prescribed drugs (Table 1), but both are associated with potentially severe pharmacokinetic interactions and toxicity. Not to mention that in Africa, some of these drugs are either prohibitively expensive (e.g., ITZ or VRC) or totally out of reach (e.g., PSZ) [61].

Some TDF, like *Coccidioides immitis* [62], *Paracoccidioides brasiliensis* [63], or *Histoplasma capsulatum* [64], have the ability to colonize surfaces and form biofilms, a phenotype that may induce high levels of resistance and enhanced virulence. Indeed, multiple strains are increasingly reported to be resistant to different antifungals [65,66,67]. Although less-publicized than antibiotic resistance, this real threat is accelerating over time [68], since most fungi have highly plastic genomes and reproduce rapidly, quickly generating variants selected for resistance. Understandably, antifungal resistance has undesirable consequences not only for human and animal health but also for forest protection and agriculture, because the already limited therapeutic options are becoming significantly reduced [69].

In the last two decades, some progress towards vaccine development against some TDF, like *Blastomyces dermatitidis*, *Coccidioides* spp., *H*. *capsulatum* [70,71], *Paracoccidioides* spp. [72], *Sporothrix* spp. [73], and *T*. *marneffei* [74], has been made using mice as a model. However, the design of safe and effective vaccines is still in its infancy due to the lack of knowledge about immunity against these fungal infections but also because of the scarcity of epidemiological data regarding the incidence and prevalence, which would allow pharmaceutical companies to decide whether human vaccines are cost-effective. For all these reasons, having reliable, affordable, and widely accessible diagnostic methods for TDF-related mycoses should be a priority for researchers in the subject and policymakers in order to improve epidemiological knowledge and surveillance. This would probably promote vaccine research but also could be useful to prevent disease, avoid incapacitating sequelae, tackle drug resistance, and, eventually, minimize mortality due to these fungal pathogens.

It is also important to note that the generalized lack of awareness about TDF-related mycoses leads to the non-use of protective means for people exposed and to the underutilization of testing, even in well-equipped centers, where the diagnosis is still mainly based on the inadequate traditional methods described next. For this and other reasons mentioned above, faster and more reliable diagnostic methods are needed in order to (1) quickly stablish the best available therapy to improve patient outcomes and increase survival by preventing pathogen dissemination, and to (2) avoid the administration of inappropriate treatments leading to antibiotic and antifungal resistance.

## 2. Diagnostic Methods

### 2.1. Culture-Based Diagnosis

Culture of clinical specimens (e.g., bone marrow, blood, urine, sputum, cerebrospinal and bronchoalveolar fluids, tissues, etc.) followed by a microscopic examination of colony morphology and sporulation pattern is the most frequently used method to diagnose endemic mycoses, such as histoplasmosis [75], blastomycosis [76], or sporotrichosis [77]. The main reason for this may be the widespread availability of affordable culture media, which makes this approach ideal for routine diagnostic laboratories. Table 2 summarizes the culture media commonly used to grow different TDF and the main genus-level morphological characters used for a preliminary diagnosis.

Following best practices, clinical specimens should be cultured in plates containing the most appropriate medium and incubated at 25–30 °C until mycelial growth is observed. For instance, samples from patients with suspected blastomycosis are often plated on Sabouraud dextrose agar (SDA) and incubated at 25–27 °C for 4 weeks, although other media, incubation periods, and temperatures can also be used. Similarly, the time required for growth of *Coccidioides* spp. ranges from 2 to 16 days [80], but the final report of negative results is usually completed at 28 days [103]. Then, to confirm thermal dimorphism, isolated colonies should be transferred to new plates containing enriched media (e.g., blood–chocolate agar, blood agar or brain heart infusion agar) that will be incubated at higher temperatures (35–37 °C). This triggers a phase conversion, allowing the observation of yeast morphology; for example, in samples from patients with paracoccidioidomycosis, one would typically observe yeasts surrounded by multiple budding daughter cells [104], while in those from patients with sporotrichosis, yeast are elongated and cigar-shaped [96]. Notably, despite careful culture maintenance, the dimorphic transition may not always occur since it depends not only on temperature but also on nutrient requirements and the physiology of each strain [105].

Culture remains the gold standard for the diagnosis of most endemic mycoses, but this method is not ideal for several reasons. First, the fastidious and slow-growing nature of TDF (see Table 2) renders it a time-consuming and labor-intensive process that delays the correct diagnosis and initiation of treatment. Second, some morphological features considerably overlap among different genera of TDF, and so culture-based diagnosis involves the subjective observation of subtle differences in colony morphology and sporulation characteristics, which is one reason why it requires well-trained staff. For example, the conidia of *Emergomyces crescens* are arranged in complex “florets” on slightly swollen stalks reminiscent of those of *Blastomyces parvus* [18], while the mycelial form of *Emergomyces africanus* closely resembles *Sporothrix schenckii* [16]. Additionally, culturing may be confusing due to contaminations, and phenotypic features can vary depending on the medium [106], which leads to misidentifications and, ultimately, to the prescription of unnecessary drugs, causing toxicity and resistance in patients [107]. Third, the overall sensitivity of culturing is relatively low. For instance, in patients with mild to moderate pulmonary histoplasmosis, cultures of respiratory specimens are often negative, and positive results may be masked due to the overgrowth of commensal organisms [75]. Moreover, some TDF, like *Paracoccidioides lobogeorgii* (formerly, *P. loboi*, and also known as *Lacazia loboi*) are unculturable [27,108]. Last but not least, culture maintenance involves a significant risk of laboratory-acquired infections, given the specially high spore load, orders of magnitude higher than in nature, which can be aerosolized accidentally [109]. Indeed, as previously mentioned, most TDF must be handled in BSL-3 facilities, which further discourages the use of culture techniques for identification of these pathogens.

### 2.2. Direct Microscopic Examination and Histopathology

A second low-cost classic approach to a presumptive diagnosis, with much less risk than cultures, is the direct microscopic visualization of the causative agent in freshly collected clinical specimens. This approach has several advantages: (i) different histochemical stains providing good staining properties with minimal background are generally available (Table 3); (ii) it enables a rapid diagnosis while waiting for cultures; and (iii) it allows complete characterization of the pathogenic phase, including the analysis of micromorphological traits and mode of reproduction. Therefore, when combined with complete clinical information, detailed histopathological studies may provide sufficient information for the correct identification of a TDF, being particularly suited when cultures are negative or unobtainable, and when dealing with very slow-growing organisms [110].

This seems to be the case for African histoplasmosis, a disease caused by *Histoplasma capsulatum* var. *duboisii*, also referred to as *H*. *duboisii*, with predominant involvement of skin and subcutaneous tissues (nodules, umbilicate papules, abscesses, ulcers, etc.) but also lymph nodes and bones.

This form of histoplasmosis is frequently diagnosed based on histopathological findings [20], in the same way that the diagnosis of sporotrichosis, a deep cutaneous mycosis, relies on histopathology combined with identification by culture [114].

However, similar to culture-based diagnosis, histopathology is also expertise-dependent, again, due to morphological similarities shared by different TDF [17]. For example, the yeast-like cells of *H*. *duboisii* can be confused with those of *Blastomyces* spp. because of their similar size and the presence of thick refractile walls [115]. Similarly, the etiologic agents of emergomycosis, *Emergomyces* spp., develop as small budding yeasts resembling those of *H*. *capsulatum* in affected tissues (although, as previously mentioned, in culture, the conidia of some *Emergomyces* species are arranged in stalked “florets” similar to those of *B*. *parvus*). Consequently, every effort should be made for an adequate differential diagnosis to exclude histoplasmosis and blastomycosis, but also tuberculosis, talaromycosis and listeriosis [76]. Indeed, only coccidioidomycosis and paracoccidioidomycosis can be relatively safely diagnosed by histopathologic identification, since both infections are characterized by the presence of easily distinguishable structures in secretions and tissues, i.e., spherules and “pilot’s wheels”, respectively (Table 2 and Table 3). In both cases, a simple, inexpensive, and reliable potassium hydroxide (KOH) preparation is particularly useful for a prompt and accurate diagnosis since it provides excellent visualization of the mentioned pathognomonic signs [79].

It is also important to take into account that histopathology sensitivity varies depending on the organism. For example, it ranges from 50% to 90% when trying to detect *Blastomyces* spp. [6], and it is low for sporotrichosis, given the scarcity of fungal elements typically found in infected tissues [96]. Moreover, appropriate tissue samples may not be easily obtained from all patients [106], which makes this approach difficult to implement.

As mentioned, identifying a specific TDF based solely on histopathology can be difficult, even impossible, given the almost complete absence of morphological singularities among TDF and the impossibility of using this method in some instances. These shortcomings paved the way for sero- and immunological tests, detailed in the next paragraph, for the diagnosis of endemic mycoses and treatment response assessments.

### 2.3. Serological and Immunological Tests

**Figure 3:**
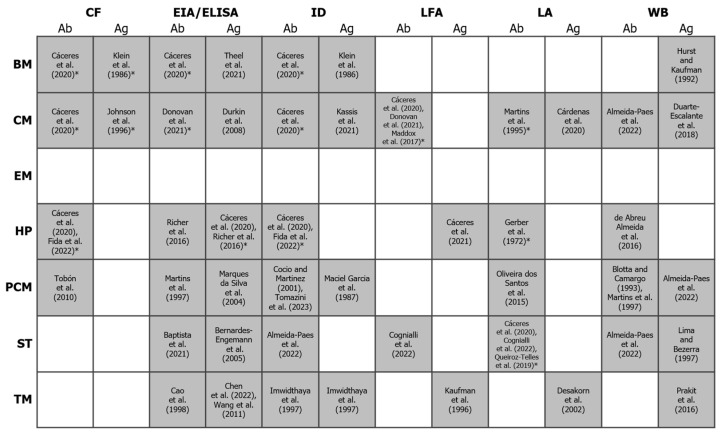
Binary heatmap showing the existence/absence of antigen and antibody detection methods (x-axis) for the diagnosis of different endemic mycoses (y-axis). Shaded cells represent the existence of a test for a given mycosis. References to studies using these methods (or citing their use) are indicated between square brackets. An asterisk indicates that at least one commercial test is available (others may exist). For more details, see the review by Cáceres and colleagues [117] and https://www.immy.com (accessed on 31 July 2024). Abbreviations: Ab = antibody; Ag = antigen; BM = blastomycosis; CF = complement fixation; CM = coccidioidomycosis; EIA = enzyme immunoassay; ELISA = enzyme-linked immunosorbent assays; EM = emergomycosis; HP = histoplasmosis; ID = immunodiffusion; LFA = lateral flow assay; LA = latex agglutination; PCM = paracoccidioidomycosis; ST = sporotrichosis; TM = talaromycosis; WB = Western blot. References in this figure: [76,117,119,120,121,122,123,124,125,126,127,128,129,130,131,132,133,134,135,136,137,138,139,140,141,142,143,144,145,146,147,148,149,150,151,152,153,154].

Cultures may become positive only late on the course of infection, and it may be difficult to obtain proper specimens for histopathology; so, alternative methods based on the detection of circulating antibodies (Ab) or antigens (Ag) in different body fluids, such as blood, urine, saliva or respiratory secretions, have been proposed (Figure 3).

Antigen tests provide direct proof of a fungal infection by reveling the presence of biological molecules produced by the pathogen (mainly proteins, but also polysaccharides or carbohydrates), such as *Blastomyces* adhesin 1 (BAD1), the surface protein adhesion WI-1 or galactomannan (*Blastomyces* spp.), coccidioidin (*Coccidioides* spp.), or gp43 (*Paracoccidioides* spp.).

As shown in Figure 3, there are several strategies for Ag testing, including immunodiffusion (ID) and tests using enzyme-labeled antibodies, i.e., enzyme immunoassays (EIA) and enzyme-linked immunosorbent assays (ELISA); the latter are also useful to detect and quantify Abs. In the case of EIAs, Ags are immobilized on a solid surface and then complexed with the Ab linked to a reporter enzyme. Antigen detection is accomplished by measuring the activity of the reporter, after an incubation with the appropriate substrate to produce a measurable product.

Urine and serum Ag EIAs designed for *H*. *capsulatum* are not able to distinguish it from its close relative *H*. *duboisii*. In contrast, ID assays effectively differentiate these species, although this type of Ag test is not widely available [116]. An in-depth explanation of these and other immunological tests is out of the scope of the present review. For this purpose, see, for example, Cáceres, DH, T Chiller and MD Lindsley [117].

What is clear is that, in immunocompetent patients, Ag tests are more likely to be positive during the early acute stage of the infection because Ag levels tend to diminish over time. In contrast, in immunocompromised patients, Ag testing is often used in early and in late disease stages, since antigens might be presents for long periods [117]. It is important to note though the wide variation in Ag production among species and isolates (for instance, *Paracoccidiodes lutzii* often produces either very low levels or no gp43, in contrast to its sister species *P. brasiliensis* [118]).

As mentioned, Ag testing can be performed on different body fluids, with urine Ag tests being particularly interesting for several reasons: (i) They are based on the use of readily available non-invasive samples. (ii) The turnaround time is relatively short (for example, in patients with blastomycosis, results are frequently available within 48 h [155]). (iii) Their sensitivity seems to be higher than that of Ag tests using serum or bronchoalveolar lavage to diagnose blastomycosis [13] or histoplasmosis [156], among other endemic mycoses, and are considered particularly advantageous for disseminated forms of histoplasmosis [157]. Indeed, urinary Ag tests represent an improvement in the management of histoplasmosis, especially in highly endemic areas, where it frequently coexists with tuberculosis [158]. (iv) They are very easy to perform. For these reasons, urine Ag tests often guide physicians on the therapy to be administered until cultures are available [33], and they seem to be a suitable option for point-of-care testing (POCT) close to the site of patient care.

Assays for the detection of Abs are also frequently used for the diagnosis of endemic mycoses. Several common testing strategies are available for Ab detection (Figure 3), including latex agglutination (LA), complement fixation (CF), and immunodiffusion (ID). LA tests are based on the ability of a specific Ab to bind to a suspension of antigen-coated polystyrene latex beads. When Abs are present in the sample, beads agglutinate and large clumps are then observed on a clear background. In contrast, when samples are negative, the suspension of beads has a milky aspect with no background clearing. Regarding CF assays, they are based on the lytic properties of the complement system, a set of proteins naturally present in human serum that react with Ag-Ab complexes. These proteins first need to be destroyed by heat and replaced by synthetic complement proteins with a known concentration. Then, the Ag of interest is added to the serum, along with an indicator system, i.e., sheep red blood cells (sRBCs) coated with anti-sRBC antibodies. When the serum sample contains an Ab against the Ag of interest, both bind together, forming Ag-Ab complexes. Complement proteins get fixed to these Ag-Ab complexes, avoiding sRBC lysis. When serum samples do not contain the Ab, the complement remains free in the mixture, and so they can fixed on the sRBC-Ab complex, which results in hemolysis of the sRBCs (the solution turns pink and the test is considered negative).

As for ID tests, these solid-phase assays are performed in an agar matrix by placing the Ag and Ab in opposing wells (generally, a known Ag is placed in a center well within the matrix and serum samples and controls are placed in surrounding wells). Reagents are allow to diffuse toward each other so, if Abs are present in the sample, a thin white line will be observed due to Ag/Ab precipitation.

Notably, the sensitivity of different types of Ab tests greatly differs. For example, compared to EIA, the sensitivity of LFA can be as low as 30% in patients with coccidioidomycosis. This seems to be directly related to the duration of illness, with subjects with positive LFA tests tending to be sick longer than those with false-negative LFA tests [123]. Despite this, the detection of Abs with different tests is still the main method for the diagnosis of coccidioidomycosis [125]. First, Ab production can occur up to six weeks after exposure in immunocompetent patients, while those with a weakened immune system are generally unable to mount an immune response. Indeed, the rate of false-negatives can be high in immunocompromised patients, but also during the acute phase because disease development can be so fast that there is no time to produce detectable Ab titers [99,159]. This could be also the case of Ab ID and Ab CF tests for the detection of *Blastomyces spp.*, which present a very low sensitivity (<45%) [160]. A second limitation of Ab testing is that distinguishing between past and current active infections is not possible because Abs may persist if patients are recurrently exposed to the fungus [131], as it might occur in endemic areas. In other words, Ab tests are mainly helpful in immunocompetent patients able to produce a quantifiable humoral response after the acute phase, when high levels of Ab are still present in the host. Only under these circumstances, Ab assays have shown acceptable sensitivity and specificity, thus representing a relatively accurate means of diagnosing endemic mycoses, especially when used in combination with Ag tests [125,131,161].

Sero- and immunological tests remain valuable assets to support endemic mycosis diagnoses, particularly when direct detection fails, as they provide faster results than culture and microscopy. Furthermore, they also offer reasonable analytical sensitivity, tending to be most useful for severely ill patients, with, presumably, a greater fungal burden [13]. In these patients, determining Ag and Ab titers before and after treatment can serve as indicator of its efficiency. Moreover, several seroimmunological tests for the diagnosis of TDF are commercially available (see, for example, [162,163] and references in Figure 3), which enables clinicians to select the best-fitting diagnostic option. However, sensitivity and specificity variations, as well as significant disparities in inter-laboratory results, have been observed among certain commercial tests [164], which evidences the need for standardization in order to minimize the effect of pre-analytical and analytical factors on their performance. Additionally, most countries where diseases caused by TDF are endemic still face production, distribution, and cost problems [93], not to mention that commercial assays are only available for the most prevalent endemic mycoses [165]. For instance, so far, no commercial or in-house Ag/Ab test has been designed for emergomycosis [61], an endemic mycosis with involvement of the skin, lungs, gastrointestinal tract, bone marrow, etc., common in immunocompromised patients. It is frequently misdiagnosed as blastomycosis or histoplasmosis [19], or even as varicella or scrofuloderma, which significantly alters treatment outcomes [166]. Commercial tests are also scarce for the diagnosis of talaromycosis, paracoccidioidomycosis, and sporotrichosis. For the latter, in particular, there used to be an Ab LA test on the market (IMMY^©^, Norman, OK, USA), whose sensitivity greatly varied from 100% in disseminated forms to 56% in cutaneous forms [145,167]. This Ab LA test seems to be no longer manufactured since it is not available on the company’s website (https://www.immy.com; last accessed on 31 July 2024).

Additionally, on the negative side, the specificity of both immunological and serological tests can be very low, and so diagnoses exclusively based only on Ag or Ab assays are often classified as “probable” or “possible cases” [117]. In this regard, it is well known that some Ags produced by different TDF (e.g., *Emergomyces* spp. and *Histoplasma* spp.) are extremely similar, which is a reason why a high degree of cross-reactivity has been observed. For instance, Ag detection via EIA in urine and serum (also in bronchoalveolar lavage and cerebrospinal fluid samples) is a fast method for a probable diagnosis of infections caused by *Blastomyces* spp., but cross-reactions with *Histoplasma* spp. [13] and *Emergomyces* spp. [166,168] are frequently reported. *Blastomyces* cross-reactivity with *T*. *marneffei*, the most important TDF causing systemic mycosis in immunocompromised patients in Southeast Asia, has also been observed in urine Ag tests [33]. Likewise, using *Histoplasma* antibody EIAs, cross-reactions have been noted in urine samples from patients with coccidioidomycosis [131].

Furthermore, some Ags, like galactomannan, are shared not only among different TDF but also with other not so closely related filamentous fungi. For instance, in patients with confirmed blastomycosis, false-positive results for *Aspergillus* galactomannan have been reported when using bronchoalveolar lavage fluid samples [169]. As can be imagined, cross-reactivity is an undesirable drawback that limits clinical use, in particular for evaluating patients suspected to have blastomycosis or histoplasmosis, since their endemicity areas greatly overlap. For this reason, these two endemic mycoses could be easily misdiagnosed if only based on sero-immunological results [75].

Ag and Ab tests still play an important role in clinical settings, but they may be of little help on their own for the diagnosis of endemic mycoses. In the case of histoplasmosis, it has been proved that the combination of Ab and Ag EIA tests improves the diagnostic accuracy [131], in a similar way that combining Ab and Ag ID assays increases the diagnostic sensitivity in patients with coccidioidomycosis [125]. Therefore, efforts should be directed to design new generations of Ab/Ag tests with enhanced sensitivity and specificity to be used in combination so they could be truly useful for the diagnosis of TDF-related mycoses and to monitor the treatment response.

The methods mentioned so far (i.e., cultures, histopathology, and Ag/Ab detection) can serve as basis for the presumptive diagnosis of different endemic mycoses. However, they all have some shortcomings (most TDF grow very slowly in culture, some species are non-culturable, others share considerable clinical and histopathological overlap, and, in some cases, clinical manifestations can even be easily confused with those of unrelated diseases). Therefore, researchers have tried to obtain diagnoses not affected by misleading positive results due to cross-reactions and false-negatives related to low sensitivity by introducing methods based on the detection of organic molecules by spectrophotometry, as explained next.

### 2.4. Matrix-Assisted Laser Desorption/Ionization–Time of Flight Mass Spectrometry (MALDI-ToF MS)

In recent years, matrix-assisted laser desorption ionization–time of flight mass spectrometry (“MALDI-ToF MS”) has become a popular alternative for pathogen detection [170], with two main systems being available, i.e., MALDI Bruker Biotyper (Bruker Daltonik GmbH, Bremen, Germany) and VITEK MS (bioMérieux, Craponne, France). Regardless of the platform, this culture-based method relies on the detection of highly abundant species-specific proteins in different clinical samples. In short, a protein spectrum is generated for each target specimen and then is used as a signature profile with peaks that are unique to the species represented in the specimen analyzed. The spectrum is compared to a reference database, and so the specimen can be successfully identified at species level if its spectrum matches with some of the reference spectra (for a further explanation, see Patel, R [171]).

To achieve reliable results, the appropriate matrix, which isolates individual proteins, protects them from breaking up, and allows desorption by laser energy [171], and an optimized sample preparation protocol need to be used, as both aspects may influence the quality and accuracy of the spectra [172].

MALDI-ToF MS has been extensively used to identify countless pathogenic bacteria, becoming an almost indispensable tool in microbiology laboratories [173]. It has been also introduced into the identification of clinically relevant yeasts (mainly *Candida* and *Cryptococcus* species), for which a cutoff value of 1.7 has been defined for optimal identification [174]. Indeed, MALDI-ToF MS has changed the diagnostic workflow at medical centers working with fungal pathogens worldwide [170], but little progress has been made with respect to TDF. The reason is that its application to TDF diagnosis is limited and subjected to a major caveat: a comprehensive curated database does not exist, since spectral data have been generated for only a few strains of some species. Specifically, the Vitek MS system includes the spectra of *B*. *dermatitidis*, *C*. *immitis*, *C*. *posadasii*, *H*. *capsulatum*, and *S*. *schenckii* [175]. It seems to be especially good at identifying different genera (100% accuracy), but its utility greatly varies depending on the species. For example, it is unclear whether it is able to distinguish between *H*. *capsulatum* and *H*. *duboisii* [116]. The same seems to occur with the other MALDI-ToF MS system (Bruker Biotype). Due to database incompleteness, this system is not able to identify the species *T*. *marneffei* [176,177]. Indeed, it fails to identify up to four members of the genera *Talaromyces* and *Penicillium* at the species level.

On the other hand, MALDI-ToF MS seems to be a robust method for *Paracoccidioides* species differentiation, with all isolates being correctly identified as *P*. *brasiliensis* or *P*. *lutzii*, with log score values of >2.0, although this result was obtained using a small in-house database [178]. There is another in-house library that includes spectra for both yeast and mycelial phases of *H*. *capsulatum* [179]. Interestingly, these spectra are completely different, probably due to differences in gene expression, which allows the identification of both morphological stages. Still, the most reliable results were obtained for mycelial phases.

Some authors claim that MALDI-ToF MS shows promise for the fast and accurate identification of pathogens causing human mycoses, including TDF [170]. However, it should be noted that, currently, this methodology is only successful using clinical isolates, and that it is not widespread among researchers working on TDF. Moreover, results are somewhat contradictory. What seems clear is that MALDI-ToF MS-based identification of the species level requires reference database expansion and refinement by obtaining spectral data from as many species, strains, and phases as possible. Equally important is that curated reference libraries become publicly available and interoperable to allow practical use.

### 2.5. PCR-Based Molecular Diagnosis

Given the already mentioned drawbacks associated with cultures, histopathology, Ag/Ab detection, and MALDI-ToF MS, in well-equipped laboratories, these approaches have lately been replaced by nucleic acid-based detection methods. Indeed, their incorporation into what has been traditionally a morphology-based discipline has revolutionized the diagnosis of countless diseases, including those caused by TDF. However, in most endemic areas, the application of molecular techniques, which are crucial for taxonomic studies, to disentangle species complexes is far from routine [165].

Two main molecular strategies are commonly used for the detection of pathogenic fungal species or varieties, i.e., polymerase chain reaction (PCR) and quantitative real-time PCR (qPCR). Standard PCR, developed by the Nobel Prize winner Kary Mullis and collaborators, allows the exponential amplification of a particular DNA fragment (generally a multi-copy gene) in vitro using a thermostable DNA polymerase and one specific primer pair [180]. If the targeted gene is present in the sample(s) analyzed, thousands to millions of gene copies are obtained after several rounds of DNA denaturation, annealing, and extension conducted in a thermocycler. Thus, using intercalating dyes, these PCR products can be detected by gel electrophoresis as fluorescent bands. In general, a negative result (absence of the expected band in agarose gels) may be sufficient to exclude a diagnosis of proven or probable endemic mycosis, while two positive PCRs would be required to confirm the diagnosis, as in other fungal diseases like aspergillosis [181].

The popularization and optimization of the original technique has led to a broad range of variants. These include, among others, (i) nested PCR (it involves two different primer pairs and the use of the PCR products of a first PCR as template for a second amplification round [180]); (ii) multiplex PCR for the simultaneous detection of several gene targets [182]; (iii) reverse transcription PCR (RT-PCR), which allows the detection of RNA, instead of DNA, starting with reverse transcription of RNA and following with DNA amplification by two different enzymes [183]; and (iv) quantitative real-time PCR (qPCR), another frequently used molecular technique that allows the detection of amplification in the exponential growth phase of the reaction [184]. This latter PCR variant is performed in a closed system and gel electrophoresis is not required after amplification, which reduces the chances for contamination and shortens the detection time. Interestingly, qPCR allows a fungal burden quantification, which is very convenient for clinical diagnosis and treatment follow up [165].

**Figure 4:**
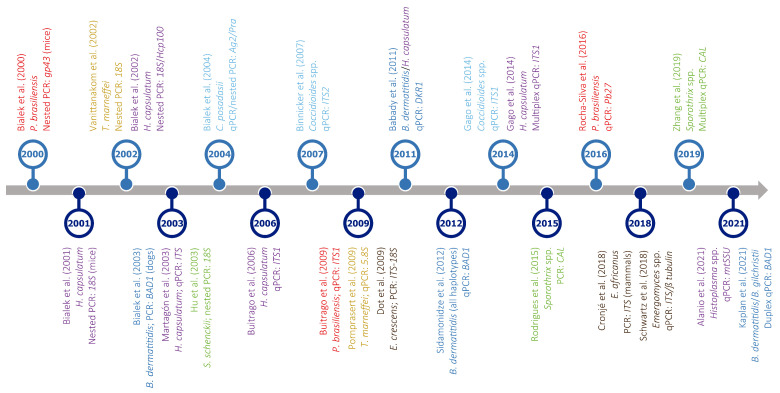
Timeline of PCR-based assays developed for the detection of different thermally dimorphic fungi. Target organisms (represented in different colors: dark blue—*Blastomyces* spp.; light blue—*Coccidioides* spp.; brown—*Emergomyces* spp.; purple—*Histoplasma* spp.; red—*Paracoccidioide*s spp.; yellow—*Talaromyces* spp.; green—*Sporothrix* spp.), type of assay (PCR, nested PCR, qPCR, or multiplex qPCR), and amplified gene(s) are indicated below each reference. For easy presentation, this graph shows only a selection of studies published in the last two decades. Most studies were based on clinical samples; otherwise, it is indicated between brackets after the gene name. References in this figure: [78,185,186,187,188,193,194,195,196,197,198,199,200,201,202,203,204,205,206,207,208,209,210,211].

PCR-based approaches offer particularly high specificity and sensitivity if the target choice and primer design are carefully performed. Indeed, they can detect very low amounts of DNA of an etiological agent in samples collected from patients with a low fungal burden [162]. Not only this, PCR and qPCR assays are useful for detecting point mutations and differentially expressed genes potentially associated with antifungal resistance, respectively [67]. Furthermore, conventional PCR and its variants are suitable for both fresh and archival paraffin-embedded clinical samples; so, they allow not only diagnosis and screening but also large-scale retrospective epidemiological studies [13,75]. Given that PCR-based assays involve the use of DNA samples, instead of highly infectious cultures, and are much faster than culturing, they are very convenient to use in well-equipped laboratories, particularly to confirm the diagnosis in cases where more than one fungal infection is possible [75]. Because of these advantages, numerous PCR-based tests have been developed in the last decades for the detection of the causative agents of most endemic mycoses (Figure 4; for an extensive review, see Valero, C, MT Martín-Gómez and MJ Buitrago [165]). These include, for example, a qPCR assay for the simultaneous detection and differentiation of *B. dermatitidis* and *H. capsulatum* [185], which are responsible for most cases of two overlapping endemic mycoses, i.e., blastomycosis and histoplasmosis, respectively.

As can be seen in Figure 4, regardless of the use of PCR or qPCR, the multi-copy nuclear ribosomal internal transcribed spacers (*ITS1* and *ITS2*) are the regions most frequently targeted. The nuclear small ribosomal subunit gene (*SSU* or *18S*) is also a common target, although others, such as *BAD1* [78], *gp43* [186], *Pb27* [187], or the mitochondrial small ribosomal subunit gene (*mtSSU*) [188], have also been used for the PCR-based diagnosis of endemic mycoses. In this regard, and despite the increasing use of PCR-based diagnosis to detect low abundance nucleic acids, there is no consensus among laboratories on which molecular region or method is best to use, even if *ITS*s are the universal DNA barcodes for the identification and taxonomical classification of fungi [189,190].

Apart from this lack of agreement, PCR-based assays have other limitations. For example, they require complex pretreatment (DNA must be extracted using expensive commercial kits) but, even so, they can be inhibited by different substances, mostly in formalin-fixed and paraffin-treated samples [191]. Likewise, no amplification can occur due to template degradation, or if the concentration of fungal DNA in clinical samples is below the detection limit [192]. That is to say, PCR and its variants are highly sensitive and specific, but not infallible. For instance, according to the results presented by O’Dowd and collaborators [160], the sensitivity of PCR assays designed for the detection of *Blastomyces* in sputum and bronchoalveolar lavage fluid ranges from very low to moderate (40–75%), respectively.

Furthermore, when using either nested PCR, which is prone to contamination, or qPCR assays on samples comprising high concentrations of DNA from closely related TDF, false-positive results can be obtained, although these are generally uncommon [103,212,213]. Additionally, it is important to note that PCR-based studies focused on the diagnosis of endemic mycoses other than histoplasmosis are relatively scarce, especially in the case of talaromycosis, emergomycosis, and blastomycosis. For example, PCR assays have been only infrequently used as an aid in the diagnosis of blastomycosis by a few laboratories [13,75], while no PCR assays have been validated for emergomycosis [61]. Moreover, there is limited availability of reagents on the market, and only a single specific PCR for the diagnosis of coccidioidomycosis is currently commercialized [201], which compromises the use of this method [165].

Still, the main disadvantage of DNA-based diagnosis, mainly if based on qPCR, is that sample processing and gene amplification have to be performed by trained personnel using sophisticated equipment, and so its use is essentially restricted to reference laboratories located in North America and Europe. Lastly, amplicons obtained by PCR should be sequenced, and newly generated sequences should be compared to credible molecular data deposited in public databases in order to confirm that no contamination occurred, which may be impractical when many clinical samples need to be screened.

Some authors envision that PCR-based diagnosis will eventually become standardized and widely available in strong health systems [13,214], but it will not easily be a routine assay in resource-limited endemic areas. Thus, molecular diagnostic approaches not relying on a generally unaffordable thermocycler may be the best solution to diagnose TDF-related infections [215].

### 2.6. Isothermal Amplification Methods

Although PCR-based methods are highly reliable for the diagnosis of endemic mycoses, they are not technically accessible for routine clinical practice, which makes it impossible to use them in POC settings. In this context, isothermal amplification methods (IAM), based on the replication of DNA at a constant temperature, could provide the most suitable and versatile alternative.

**Figure 5:**
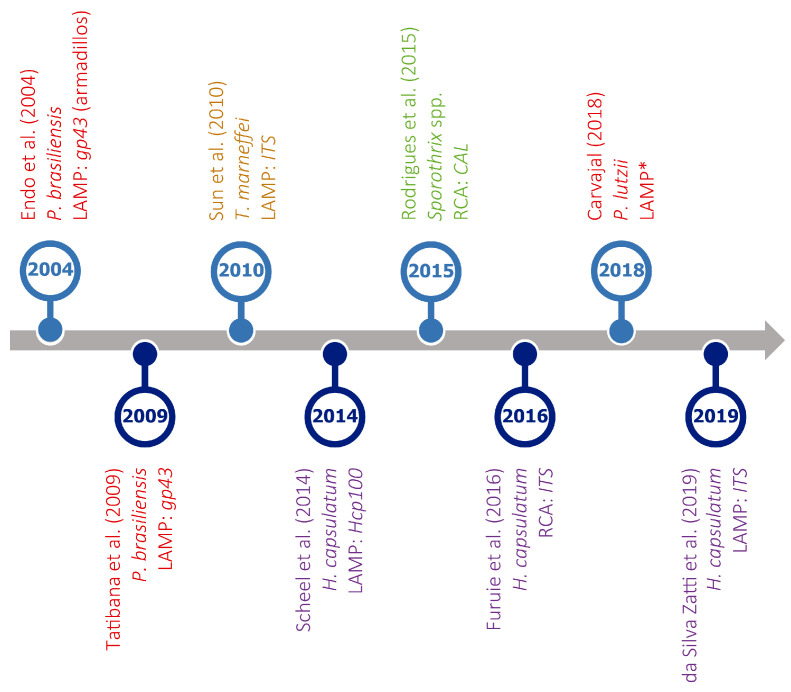
Timeline of isothermal amplification assays developed for the detection of different thermally dimorphic fungi. Target organisms (represented in different colors), type of assay (LAMP or RCA), and amplified genes are indicated for each reference. * No information available. References in this figure: [217,218,219,220,221,222,223,224].

A number of methods for overcoming PCR-temperature dependence are currently available, including, among others, nucleic acid sequence-based amplification (NASBA), self-sustained sequence replication (SSR), strand displacement amplification (SDA), rolling circle amplification (RCA), and loop-mediated amplification (LAMP). For a review of IAM for the detection of pathogenic fungi see da Silva Zatti and colleagues [216]. However, only LAMP and RCA have been used to diagnose endemic mycoses (Figure 5), and the first is the most popular isothermal method.

LAMP assays are based on the use of a thermophilic polymerase obtained from *Bacillus stearothermophilus (Bst* polymerase) and two specific primer sets [225]. These four primers bind to six different regions of the target (ideally a multi-copy gene to increase sensitivity without compromising specificity), which then is amplified at a constant temperature (60–65 °C). Additionally, a third primer set, the so-called loop primers, was introduced to further accelerate the amplification reaction [226]. The molecular mechanism behind LAMP is very complex, but the method is very easy to use, fast, and cost-effective because the *Bst* polymerase is not only highly resistant to inhibitors [227] but also cheaper than the enzyme traditionally used for PCR, *Taq* polymerase [228]. Furthermore, the amplification and detection of the gene(s) of interest can be completed in a single step, and the results can be directly read with the naked eye (color or turbidity changes are easily observable in the reaction tubes) or by real-time fluorescence, without gel electrophoresis. These are great advantages that could open new research avenues for fungal diagnosis. Indeed, LAMP could potentially be used in low-resource settings, such as endemic areas of mycoses caused by TDF, where the diagnosis is still primarily based on cultures.

LAMP has been widely applied to diagnose various tropical diseases caused by viruses [229,230,231], bacteria [232], protozoa [233,234,235], or helminths [236,237], offering good results. It has also been used for the diagnosis of mycoses caused by fungi not closely related to TDF (Table S3), and has been proposed as one of the best alternatives to classical PCR-based approaches, due to its simplicity, the low costs associated, and its relatively high sensitivity and specificity [216,238].

However, LAMP has not been sufficiently explored for the diagnosis of endemic mycoses, as indicated by the low number of assays currently available (Figure 5), often including a limited number of clinical specimens.

Focusing on the diagnosis of histoplasmosis, the most prevalent endemic mycosis worldwide, two LAMP assays have been designed (Table S4). Scheel and colleagues [220] developed a LAMP assay based on a single-copy gene (*Hcp100*), proved specific under laboratory conditions (results were negative in all healthy controls). Notably, the specificity of this assay was determined using DNA extracted from other fungi, but only some of them were closely related to *Histoplasma*, and no DNA samples of *Emergomyces*, its sister genus, and *T*. *marneffei*, a species causing symptoms very similar to those of histoplasmosis, were included (Table S4). Moreover, the sensitivity of the *Hcp100* LAMP assay was not high enough, as it only detected the target in 67% of antigen-positive urine specimens. The presence of inhibitors as a cause of the decreased sensitivity was ruled out using urine samples spiked with known concentrations of *H*. *capsulatum* DNA (all were found positive). This suggested that DNA degradation may be responsible for the low sensitivity reported, although the fact that *Hcp100* is a single-copy gene could also have contributed to some detection errors. Despite being a pioneer study, the authors acknowledged that further evaluation using additional fresh-frozen urine, serum, and blood samples is required to validate their test.

The second *Histoplasma* LAMP assay currently available was designed to amplify the *ITS* region [224]. In this study, 26 bone marrow specimens (which are much more difficult to obtain than urine, sputum, or blood samples) from HIV/AIDS patients with symptoms of progressive disseminated histoplasmosis were used. Only 11 of these samples corresponded to patients with positive cultures for *Histoplasma* spp. Additionally, one blood sample from a patient suspected to have histoplasmosis and another five from healthy individuals were also included (Table S4). Of interest, the authors analyzed a heparinized blood sample spiked with *H*. *capsulatum* yeasts directly, with no previous DNA extraction, which represents a huge advantage for molecular diagnosis. However, when using positive cultures as reference (that is, considering only the mentioned 11 bone marrow samples), the *ITS* LAMP assay showed only 54% sensitivity and a specificity of 95%. Using *Hcp100* as reference, the test reached 83% sensitivity and 92% specificity, which are still limited values.

The sensitivity of the *Histoplasma* LAMP assays just mentioned is far from 100%. It is also important to note that none of these studies included more than one sample of the African lineage (Table S4), which could have influenced their results.

Other than these, LAMP assays have been designed for *Paracoccidioides* spp. [217,218,223] and *T*. *marneffei* [219], but, to the best of our knowledge, none are currently available for the diagnosis of potentially fatal diseases caused by species of *Blastomyces*, *Coccidioides*, *Emergomyces*, and *Sporothrix*. Therefore, their design and optimization should be a first priority for researchers working on the topic.

There is no doubt that LAMP has great utility and practical value for the diagnosis of endemic mycoses because of its high sensitivity, short turnaround time, and its potential for implementation in POCT. Nevertheless, it also has obvious disadvantages, such as strict and complex primer design and high rate of false-positives due to primer dimers [239,240], especially with long incubation times that may be necessary to amplify fungal DNA, according to the conditions reported for both *Histoplasma* LAMP assays [220,224]. In fact, it is well-known that LAMP assays are prone to non-specific amplifications and contamination, and so extremely careful procedures are required, including separate handling of DNA samples and reagents [241]. Considering this, the emphasis should be placed on the analysis of further data and the optimization of LAMP assays before the use of this IAM becomes popular among clinicians.

Several alternatives have been proposed to enhance LAMP analytical specificity by decreasing the probability of primer dimer formation. These include the use of species-specific primers with a highly controlled design [240], fluorescent probes [242], touchdown protocols [243], or the addition of chemical compounds to the reaction mixture to stabilize the structure of the primers and prevent the formation of dimers [244,245]. Even so, a new type of IAM, called stem-loop-primer-assisted isothermal amplification (SPA), has been recently developed using viral DNA as target [246]. Compared to conventional LAMP, SPA seems to offer a reduced risk of false-positives because it relies on a highly simplified primer design that provides ultralow background amplification. If this holds true when applied to fungal DNA amplification, SPA could be highly advantageous to perform disease screening and diagnosis in POC settings.

RCA, an IAM described by Fire, A and SQ Xu [247], relies on specially designed oligonucleotides whose ends are complementary to the target, known as padlock probes. To mimic bacterial plasmid amplification, RCA also depends on two enzymes, i.e., one thermostable polymerase, such as phi29 or *Bst*, and one ligase, like the T4 DNA ligase, used during the amplification reaction for strand displacement and padlock probe circularization, respectively. In the presence of dNTPs and only two primers, which makes RCA primer design much more flexible and simpler than that of LAMP primers [248], padlock probes hybridize to the target, enabling probe ends to be joined by the ligase and thus forming circular DNA molecules. These are amplified by an incubation at a constant temperature, giving rise to a large amount of DNA that can be visualized by fluorescence or colorimetric approaches [249]. Given the simplicity, sensibility, and robustness of this gel-free technology, and its ability to detect species-specific polymorphisms, the authors have claimed that RCA should be a routine molecular test for fungal diagnostics in low-income regions [189,221]. Indeed, this IAM is increasingly used to diagnose pathogenic fungi, including some species of the genera *Aspergillus*, *Cryptococcus*, *Exophiala*, *Fonsecaea,* and *Trichophyton* [189,222,250].

Nevertheless, as shown in Figure 5 and Table S4, RCA has been only occasionally applied to the diagnosis of TDF-related diseases, with only two assays being available. For the first RCA assay ever presented to diagnose an endemic mycosis, sporotrichosis specifically, six species-specific padlock probes targeting single nucleotide polymorphisms in the gene encoding calmodulin were developed [221]. Using DNA extracted from pure cultures, but also from complex environmental samples, the authors found no cross-reactivity with closely related species. The second RCA assay was designed to detect different *ITS* fragments in clinical samples from patients with histoplasmosis using two padlock probes, i.e., HcPL1 and HcPL2 [222]. Although the data on sensitivity and specificity are not detailed enough, HcPL2 seems to be effective for the specific identification of *Histoplasma* spp., but HcPL1 cross-reacted with the other fungi, an issue previously reported [189]. Future improvements to current probe design could address this issue, but its occurrence along with the limited number of studies based on RCA warrants further investigation to optimize this method and to reach a deeper understanding of its potential for endemic mycosis diagnoses.

The advent of IAM has allowed equipment simplification and a visual interpretation of results, has improved the speed of diagnosis, and has decreased the amplification costs, although few companies produce the reagents required [249]. Nevertheless, these methods may be limited to some extent by false-positives and low sensitivity. Some of them, in particular LAMP, are associated with difficult primer design. Therefore, there is an urgent need to demonstrate the applicability of optimized LAMP and RCA assays as user-friendly, fast, affordable, reliable, sensitive, and deliverable methods for the molecular identification TDF in a variety of human samples.

## 3. Concluding Remarks and Future Directions

Among the more than five millions of fungal species that probably exist [251], only a few species of the order Onygenales, plus the genus *Sporothrix* and *T*. *marneffei* are capable of undergoing a temperature-dependent morphological transformation and causing the so-called endemic mycoses in humans. These thermally dimorphic fungi have attracted scientists’ attention since their discovery, not only because of their striking morphological change but also because of the increasing number of infections caused. They often affect the lungs but can also disseminate hematogenously, causing disease in virtually any organ or tissue, with symptoms being similar among different endemic mycoses.

Treatments include similar drug combinations, although there are differences in terms of the drug, dosage, and duration. For example, FLZ should be avoided to treat emergomycosis because *Emergomyces* spp. are resistant to this antifungal, as indicated by the high minimum inhibitory concentrations observed [18]. In addition, because of its high toxicity and since, among other things, azole is known to cause hepatitis, it should not be administered to pregnant women because of possible teratogenicity [6]. Despite this, when WHO-listed essential systemic antifungal drugs (AmB, ITZ, VRC, and flucytosine) are not available, FLZ is frequently used to treat African histoplasmosis cases [20]. On the other hand, AmB, which is also highly toxic, is still considered a suitable treatment option for most TDF-related mycoses. However, its use is not recommended to treat non-life-threatening lymphocutaneous cases of sporotrichosis, also due to its side effects, such as nephro- and hepatotoxicity, hypokalemia, phlebitis, or anemia [252], and inconvenient administration [18]. Most importantly, the clinical manifestations of these mycoses can also mimic those of unrelated bacterial and viral diseases, and so an accurate diagnosis is vital to avoid increased morbidity, mortality, and antibiotic/antiviral resistance.

As mentioned in the first section, no human vaccines are available against TDF, so using optimal diagnostic methods is vital to improve epidemiological knowledge and surveillance systems, which would help to prevent and treat infections. Traditionally, the diagnosis of endemic mycoses has primarily relied on conventional methods, such as culture, serology, and histopathology, coupled with detailed knowledge of the patient’s history of exposure to the etiologic agents [75]. However, in the last decades, more advanced methodologies, including MALDI-ToF MS and molecular tests, have been developed. Given the wide range of methods, the choice of the most appropriate depends on the local epidemiology, treatment guidelines, and, overall, on its availability.

Currently, reliable PCR-based tests can only be implemented in high-income countries (even so, tests for some species are still under development or standardization), while conventional diagnostic techniques, which are not exempt from limitations, are used in routine clinical practice in most laboratories, including those located in endemic areas. Therefore, the development of an affordable, accessible, and reliable diagnostic tool for different endemic mycoses should be prioritized to allow a precise diagnosis in endemic areas, which would also improve epidemiological surveillance.

There are many unresolved research questions about the disease burden and geographic ranges, both remaining highly speculative and underestimated, given the sporadic reports of cases from poor endemic areas (particularly Africa) with no or a very limited diagnostic capacity. In this context, novel isothermal assays, currently under development in our laboratory, hold promise for improved sensitivity and specificity. These include the analysis of multiple samples from geographically distant origins, DNA extraction simplification, and the selection of a target offering better results in terms of reliability, reaction time, sensibility, specificity, etc. Nevertheless, given the possibility of detecting false-positives derived from non-specific amplifications, including signals due to primer dimers, different enhancement strategies are currently being tested for performance. The reduction of false-positives in optimized LAMP assays will increase their specificity, eventually helping to minimize mortality due to endemic mycoses.

Until these much-needed molecular tests are validated in large clinical studies and are widely available in centers with restricted resources and/or no technical expertise, the best diagnostic option seems to be a multifaceted approach combining the use of several of the methods included in this review.

## Figures and Tables

**Table 1:** Symptoms of endemic fungal diseases produced by clinically relevant thermally dimorphic fungi of the orders Onygenales, Ophiostomatales and Eurotiales.

Order (Family)	Species	Disease (Common Name)	Main Route of Infection	Phase Transition	Clinical Manifestations			First-Line Therapy// Other Treatments	References
					Cutaneous	Pulmonary	Systemic		
Onygenales (Ajellomycetaceae)	*Blastomyces* *dermatitidis* (T)	Blastomycosis (Gilchrist’s disease)	Incidental inhalation of airborne conidia or hyphal fragments released from saprophytic mycelia growing in soil. Primary cutaneous cases by skin inoculation occasionally reported.	Multicellular hyphae to unicellular yeasts, adiaspores in the case of *B. parvus*.	Solitary or multifocal verrucous nodules with irregular borders, ulcers.	Fatigue, fever, chest pain, cough, night sweats.	Meningeal signs, focal neurologic deficits, osteoarticular signs, bone pain (especially long bones and vertebrae), prostatitis (♂), adnexal pain (♀).	If not severe, ITZ for 6–12 months. Otherwise, L-AmB for 1–6 weeks, followed by ITZ (6–12 months, or long-life therapy)// FLZ and VRC.	[6,7,8,9,10,11,12,13]
	*B. emzantsi*						Osteoarticular signs.		
	*B. gilchristii*						Meningeal signs, focal neurologic deficits, bone pain (long bones and vertebrae), prostatitis (♂), adnexal pain (♀).		
	*B. helicus* ^1^						Pain (long bones or vertebrae), osteoarticular signs, prostatitis (♂), adnexal pain (♀), meningeal signs, focal neurologic deficits.		
	*B. parvus* ^2^	Adiaspiromycosis				Adiaspores associated with granulomatous lesions (host response leads to granulomas formation).	Fatigue, fever, cough, night sweats, pain (long bones or vertebrae), osteoarticular and meningeal signs; prostatitis (♂), adnexal pain (♀), focal neurologic deficits.		
	*B. percursus*	Blastomycosis (Gilchrist’s disease)				Fatigue, fever, chest pain, cough, night sweats.			
	*Emergomyces* *pasteurianus* ^3^ (T)	Emergomycosis (disseminated emmonsiosis)	Incidental inhalation of airborne conidia released from soil mycelia (not confirmed but presumed).	Multicellular hyphae to budding yeast cells, adiaspores in the case of *E. crescens.*	Plaques, ulcers and erythematous papules.	Multiple granulomatous lesions.	Fever, weight loss, anemia.	No treatment guidelines are available, but L-AmB (1–2 weeks) is recommended over FLZ. Only seven African countries have access to AmB.	[3,7,8,11,14,15,16,17,18,19]
	*E. africanus*				Erythematous papules and ulcerated and crusted plaques with or without scales.	Diffuse reticulonodular disease, consolidation, effusions, and/or lymphadenopathy.			
	*E. canadensis*				Erythematous papules and plaques, ulcers.	Solitary lung nodules.	Fever, anemia, elevated liver enzymes, and weight loss.		
	*E. crescens* ^4^	Adiaspiromycosis			Mainly restricted to lungs. Occasionally, regional lymph nodes.	Granulomatous lesions (presence of adiaspores).	It normally does not disseminate. Ocular and systemic forms only occasionally described.		
	*E. europaeus*	Emergomycosis (disseminated emmonsiosis)			Erythematous papules, plaques, and ulcers (un-common in *E. europaeus*).	Multiple granulomatous lesions.	Fever, weight loss, anemia.		
	*E. orientalis*								
Onygenales (Ajellomycetaceae)	*Histoplasma* *capsulatum* var. *capsulatum* (T)	Histoplasmosis (Darling’s disease)	Incidental inhalation of airborne microconidia released from soil mycelia.	Multicellular hyphae to unicellular yeasts.	Nodules, cellulitis, papules, necrotizing fasciitis.	Fever, cough, dyspnea, chest pain.	Fatigue, weight loss, meningeal signs, hepatosplenomegaly, shock.	ITZ (6–12 weeks to 2 years). AmB-d or L-AmB, mPRED for severe forms//PSZ, VRC or ISZ.	[8,10,11,20,21]
	*H. capsulatum* var. *duboisii*	African histoplasmosis						AmB// ITZ, FLZ and surgery.	
	*H. mississipiense*	Histoplasmosis (Darling’s disease)						ITZ (6–12 weeks to 2 years). AmB-d or L-AmB, mPRED for severe forms//PSZ, VRC or ISZ.	
	*H. ohiense*								
	*H. suramericanum*								
	*Paracoccidioides* *brasiliensis* (T)	Paracoccidioidomycosis (South American blastomycosis)	Incidental inhalation of airborne conidia (arthroconidia and microconidia) or hyphal fragments, released from soil mycelia.	Multicellular hyphae to unicellular yeasts.	Erythematous papules and nodules to ulcers. Rarer forms (sarcoidosis-like) have lichenoid appearance.	Granulomatous pulmonary disease.	Fever, lymphadenopathy, hepatosplenomegaly, anemia, eosinophilia, weight loss.	ITZ, AmB-d, L-AmB, or TMP-SMX for severe cases//VRC. Corticosteroids should be considered.	[8,10,11,22,23,24,25,26,27]
	*P. americana*								
	*P. lobogeorgii* ^5^	Lobomycosis (Lôbo’s disease)			Chronic keloidal lesions starting from the pinna of the ear, and plaques.		Only occasionally.	Clofazimine, cryosurgery for limited lesions, wide surgical excision//TMP-SMX.	
	*P. lutzii*	Paracoccidioidomycosis (South American blastomycosis)			Erythematous papules and nodules to ulcers. Rarer forms (sarcoidosis-like) with lichenoid appearance.		Fever, lymphadenopathy, hepatosplenomegaly, anemia, eosinophilia, weight loss.	ITZ, AmB-d, L-AmB, or TMP-SMX for severe cases//VRC. Corticosteroids should be considered.	
	*P. restrepoana* *								
	*P. venezuelensis*								
Onygenales (Onygenaceae)	*Coccidioides* *immitis* (T)	Coccidioidomycosis (San Joaquín Valley fever)	Incidental inhalation of airborne arthroconidia, released from soil mycelia.	Spores to spherules.	Skin nodules and ulcers, multifocal (umbilicated) papules/nodules.	Fatigue, fever, cough, dyspnea.	Headache, myalgia, meningeal signs, inflammatory arthritis.	FCZ (osteoarticular). L-AmB plus FCZ, for severe disease//ITZ.	[8,10,11,28,29]
	*C. posadasii*								
Ophiostomatales (Ophiostomataceae)	*Sporothrix* *schenckii* (T)	Sporotrichosis (rose gardener’s disease)	Skin inoculation through superficial wounds (cuts or scrapes) getting in contact to infected material. Also, by the bite or scratch of infected cat. More rarely, by inhalation.	Multicellular hyphae to unicellular yeasts.	Erythematous ulcers in a lymphocutaneous pattern, subcutaneous nodules or abscesses.	Fever, cough, dyspnea, chest pain, pulmonary cavitation.	Fever, confusion, headache, ocular lesions, encephalitis, meningitis, inflammatory oligoarthritis.	ITZ (2–12 months, depending on severity and presentation), L-AmB or AmB-d followed by ITZ for severe cases// Supersaturated solution of potassium iodide, terbinafine, FCZ. Cryosurgery for patients with severe keratotic lesions or pregnants.	[8,10,11,30]
	*S. brasiliensis*								
	*S. globosa*								
	*S. luriei*								
Eurotiales (Trichocomaceae)	*Talaromyces marneffei ^6^*	Talaromycosis	Incidental inhalation of airborne conidia and skin inoculation.		Small, umbilicated (painless) papules on face, extremities, palate; chronic genital sore.	Fever, cough, dyspnea.	Malaise, weight loss, lymphadenopathy, hepatosplenomegaly.	L-AmB or AmB-d (1–2 weeks) followed by ITZ// VRC (12 weeks).	[8,10,11,22,31,32,33]

Species are ordered by family and then alphabetically (except for the type of the genus (T), appearing first). Synonymous species names: ^1^ *Emmonsia helica*, ^2^ *Emmonsia parva*, ^3^ *Emmonsia pasteuriana*, ^4^ *Emmonsia crescens*, ^5^ *P. loboi* and *Lacazia loboi*, ^6^ *Penicillium marneffei*. * Published as “*P. restrepiensis*”. Treatment abbreviations: AmB-d = amphotericin B deoxycholate; FLZ = fluconazole; ISZ = isavuconazole; ITZ = itraconazole; L-AmB = liposomal amphotericin B; mPRED = methylprednisolone; PSZ = posaconazole; TMP-SMX = trimethoprim/sulfamethoxazole; VRC = voriconazole.

**Table 2:** Different culture media used to grow TDF and main genus-level morphological characters useful for a diagnosis.

Taxa	Culture Media	Hyphal Growth Conditions	Macroscopic Features *		Microscopic Features *		References
			Mold (25–30 °C)	Yeast (35–37 °C)	Mold (25–30 °C)	Yeast (35–37 °C)	
*Blastomyces*	BHI SDA YPD	25–27 °C, 4 weeks.	(SDA) White to whitish, cottony to glabrous colonies, gray or brown with age. Beige reverse.	(BHI) Smooth, shiny, white or cream to pinkish-brown colonies.	Delicate, short, septate, hyaline hyphae. Short or long conidiophores with oval one-celled conidia.	Large, unipolar or bipolar, broad-based budding cells.	[3,13,78]
*Coccidioides*	BA BHI MYA MYC PDA SDA	25–28 °C, 2 days to up to 6 weeks.	(SDA) Apiculated, glabrous, white colonies, when young. As they mature, they become velvety or cottony and cream- or gray-colored.	* In vitro reversion of the mold to yeast phase is not routinely performed (it requires high concentrations of CO_2_ in a rich medium and involves a high risk of infection).	Hyaline septate hyphae and barrel-shaped arthroconidia alternating with empty disjunctor cells (lacking cytoplasmatic material), responsible for their easy aerial propagation.	Large (20–200 μm in diam.), rounded, thick structures known as “spherules”, comprising a large number of small endospores (2–4 μm in diam.).	[56,79,80,81,82,83,84]
*Emergomyces*	BHI MEA PDA SBA SDA	24–30 °C, 1 to up to 4 weeks.	(SDA) Yellowish-white to pale brown or beige colonies. Tanned reverse. Glabrous, becoming powdery, slightly raised, and furrowed. Up to 3.5 cm diam.	(BHI) Small, cream to grey-brown, wrinkled, yeast-like colonies.	Hyaline septate hyphae (1–2.5 μm diam.). Conidiophores bearing a varying number of conidia (e.g., 2–4 in *E. africanus*, 1–3 in *E. pasteurianus*).	Small (2 μm to 4 μm), oval, ellipsoidal, thin-walled cells budding at narrow bases.	[3,18,56,85,86,87]
*Histoplasma*	BHI MYA MYC SDA	25 °C, 6 to 12 weeks.	(SDA) White, cottony colonies, becoming brownish with age. Pale yellow reverse.	(BHI) Smooth, moist, round, white colonies.	Septate, hyaline hypha with smooth-walled spherical to pyriform tuberculate macro (7–15 μm) and microconidia (2–5 μm).	Small or large, narrow-budding ovoid cells.	[87,88,89,90]
*Paracoccidioides*	BHI MYA MYC SDA YPD	25–30 °C, at least 4 weeks.	Flat, glabrous, white to beige-brownish colonies, sometimes wrinkled, with a few tufts of aerial mycelium.	(SDA) Yeast colonies variable in size, cerebriform, cream-colored (white to brown) and generally folded.	Septate, thin and freely branching hyphae, 1–3 μm in width, with the appearance of interwoven threads. Occasionally, chlamydospores (15–30 μm) can be observed.	Varying size (2–30 μm in diam.), oval or irregular yeasts displaying the characteristic “pilot’s wheel” configuration.	[91,92,93,94]
*Sporothrix*	BHI MYA MYC PDA SDA YPD	25–30 °C, 1 to 4 weeks.	(SDA) Smooth, creamy or pale orange to grey-orange, becoming brown or nearly black in the center. Pale yellow reverse.	(BHI) Moist, creamy or white to tan colonies, colonies with a smooth surface and round margin.	Delicate, hyaline septate hyphae (1–3 μm in diam.), usually branched. Subglobose to ellipsoidal and slightly pigmented conidia, forming a rosette at the end of erect conidiophores, plus sessile, pigmented conidia.	Colonies formed by single or multiple budding, small (2–4 × 3–6 μm), round or oval cigar-shaped yeast cells.	[56,87,95,96,97,98,99,100]
*Talaromyces marneffei* **	BHIA SDA	25–30 °C (ca. 1 week)	(SDA) White mycelium turning grayish-pink to green after sporulation with red diffusible pigment production.	Cerebriform or smooth, beige, glabrous colonies of yeasts, appearing heaped and wrinkled due to the overproduction of arthroconidia.	Conidiophore-bearing compact biverticillate penicillia composed of four or five metulae with smooth-walled conidia.	Cylindrical, ellipsoidal to rectangular yeasts, (2) 3–6 μm, with a central transverse septum. *H. capsulatum* yeast appear similar but they divide by budding rather than by fission.	[76,101,102]

Taxa are ordered alphabetically. Abbreviations: BA = blood agar; BHI = brain heart infusion; BHIA = brain heart infusion agar; MEA = malt extract agar; MYA = mycobiotic agar; MYC = mycosel agar; SDA = Sabouraud dextrose agar; SBA = sheep blood agar; PDA = potato dextrose agar; YPD = yeast extract peptone dextrose agar. * Phenotypic features can vary depending on the culture medium. ** Morphological traits at the species level, when using the medium indicated between brackets.

**Table 3:** Stains used for the diagnosis of endemic mycoses and main morphological traits at the genus level.

Taxa	Clinical Sample(s)	Stain(s)	Histopathological Findings [Confounding Organisms]	References
*Blastomyces*	Bronchoscopy samples, sputum, and exudates	CFW, Gram, KOH	Large (8–15 μm), multinucleated, round to oval yeasts, with broad-based buds often attaining the same size as the parent cells before detachment. [*Candida* spp., *Cryptococcus neoformans*, *T. marneffei* and endospores of *Coccidioides* spp.]	[87,99,111]
	Tissues	GMS, PAS		
*Coccidioides*	Sputum, bronchoalveolar lavage fluid, and pus	CFW, KOH	Tuberculoid granuloma with large (60–100 μm), thick-walled spherules filled with endospores of different sizes (2–5 μm). [*Blastomyces* spp., *Prototheca wickerhamii* and *Rhinosporidium seeberi*]	[99,111]
	Tissues	GMS, H&E, PAS		
*Emergomyces*	Blood and bronchial and cerebrospinal fluids	PAS	Small (2–5 (7) μm), ovoidal to spherical, narrow-based budding yeasts, with cells with surrounding inflammatory signs. [*Histoplasma* spp. and *Sporothrix* spp.]	[15,17,86,87,112]
	Tissues	GMS, H&E, PAS		
*Histoplasma*	Blood, bone marrow (rarely, sputum and other respiratory fluids)	Giemsa, Wright’s stain	Generally small (2–4 (6) μm), ovoid, thin- or thick-walled narrow-based budding yeasts, within macrophages or free in the tissues. Much larger (10–15 μm) in *H. duboisii*. [*Emergomyces* spp., *Blastomyces* spp., *Candida* spp., *Cryptococcus neoformans*, *T. marneffei, Trypanosoma* spp. and *Leishmania* spp.]	[87,99,111]
	Tissues	GMS, PAS		
*Paracoccidioides*	Sputum, bronchoalveolar lavage fluid, and pus	CFW, Gram, KOH, LCB, MB	Translucent, refracting, thick-walled yeasts surrounded by multiple peripheral buds (“pilot’s wheel” morphology), plus prominent intracytoplasmatic vacuoles. [*B. dermatitidis*, *H. capsulatum* and *Cryptococcus neoformans*]	[91,99,111]
	Tissues	GMS, H&E, PAS		
*Sporothrix*	Sputum, pus, blood, synovial and cerebrospinal fluids, and skin exudates	Giemsa, Gram, KOH, PAS	Asteroid bodies (eosinophil material surrounding the fungal cell, probably host immunoglobulins attached to the yeast wall). The budding yeasts are globose, cigar-shaped, or oval to spherical. [*Leishmania* spp.]	[96,98,99,113]
	Tissues	GMS, H&E, PAS		
Talaromyces marneffei *	Blood, sputum, bone marrow	Wright’s stain	Numerous binary fission yeasts (within histiocytes or extracellularly), with a central transverse septum. [*Candida* spp., *Cryptococcus neoformans*, *Histoplasma* spp., *Blastomyces* spp. and endospores of *Coccidioides* spp.]	[99,101,102,111]
	Tissues	GMS, PAS		

Taxa are ordered alphabetically. * Morphological traits at the species level. Abbreviations: CFW = calcofluor white; GMS = Grocott–Gomori methenamine silver; H&E = hematoxylin and eosin; KOH = potassium hydroxide; LCB = lactophenol cotton blue; MB = methylene blue; PAS = periodic acid–Schiff.

## Data Availability

Data supporting this review are available within the manuscript itself and in the studies referenced in the main manuscript and the Supplementary Materials.

## Supplementary Materials

The following supporting information can be downloaded at https://www.mdpi.com/article/10.3390/jof10090637/s1: Table S1. Data on selected dimorphic fungal pathogens affecting plants and animals. Abbreviations: Temp. = temperature; E = entomopathogen; H = human pathogen; M = mammal pathogen; P= phytopathogen; Z = zoopathogen; (?) = doubtful trigger. Table S2. Genes involved in virulence and or immune-evasion strategies in different TDF. Only genes with known function have been included. Note that some genes are present in different TDF, which gives an idea of their importance for virulence. Table S3. LAMP assays for pathogenic and food/drink spoilage fungi (not necessarily dimorphic fungi). Species are ordered first by taxonomic order and then alphabetically. Abbreviations: *Acl1* = ATP citrate lyase subunit 1; *amy1* = alpha-amylase 1; *anxC4* = annexin C4; *β-D glucan* = (1→3)-β-D-glucan; *βTUB* = β-tubulin; *CAP* = capsule-associated gene; *CHS-1* = chitin synthase 1; *COX* = mitochondrial cytochrome c oxidase; *cyp51A* = cyp51A gene promoter region; *EF-1α* = elongation factor 1 alpha; *gaoA* = galactose oxidase; *Hyd* = hydrophobin; *IGS1* = intergenic spacer 1; *ITS* = internal transcribed spacer; *LSU* = nuclear large ribosomal subunit; *MAT* = mating type gene; *mtSSU* = mitochondrial small ribosomal subunit; *PKS* = calmodulin or polyketide synthase; rDNA = ribosomal operon (*SSU-ITS1-5.8S-ITS2-LSU*); *SSU* = nuclear small ribosomal subunit. * Former species name: ^0^ *Exophiala jeanselmei*; ^1^ *Phomopsis phaseoli*; ^2^ *Pseudallescheria boydii*; ^3^ *Pseudallescheria boydii* and *Petriellidium boydii*; and ^4^ *Pneumocystis carinii*. ** Sample origin: A = animal; H = human (clinical); P = plant; O = other origin (food or drink). If applicable, either the name of the disease caused by the particular fungus and/or the specific animal or plant affected appears between brackets. Table S4. Summary of LAMP and RCA assays designed so far for the diagnosis of endemic mycoses. Taxa are ordered alphabetically. Those closely related to the genera *Histoplasma* and *Sporothrix* are in bold. Abbreviations: HP = histoplasmosis; NS = not specified; SP = sporotrichosis; SPC = specificity; ST = sensitivity. *Twenty-six bone marrow samples were included, but only eleven corresponded to patients with positive cultures.
