# Supplementary material for: Diagnosis of Human Endemic Mycoses Caused by Thermally Dimorphic Fungi: From Classical to Molecular Methods
Source: J Fungi (Basel). 2024 Sep 6;10(9):637. doi: 10.3390/jof10090637 (PMC11432851; doi:10.3390/jof10090637)
Supplement: Supplementary file 1 [file jof-10-00637-s001.zip › jof-3167471-supplementary.pdf]

Table S1. Data on selected dimorphic fungal pathogens affecting plants and animals.

Order	Species	Disease name	Type of pathogen (preferential host)	Phase transition trigger(s)	Reference
Capnodiales	*Hortaea werneckii*	Tinea nigra	H	Temp., nutrition, inoculum size.	[1]
	Mycosphaerella graminicola	-	P (wheat)	Starvation.	[2]
	Zymoseptoria tritici	Septoria leaf blotch**		N_2 starvation, light (?).	[3, 4]
Chaetothyriales	*Magnaporthe oryzae*	Rice blast	P (cereal crops)	Light when grown on Prune-agar medium with C and N_2.	[5]
Exobasidiales	*Meira miltonrushii*	Smut	P (leaves of *Magnolia grandiflora*)	C sources.	[3, 6]
Glomerellales	*Verticillium albo-atrum*	Vascular wilt disease	P	Culture agitation, inoculum density.	[2]
	Verticillium dahliae		P (crops)		
Hypocreales	*Beauveria bassinia**	White muscardine	Z (silkworms)	Insect hemolymph.	[1]
	Metarhizium anisopliae	Green muscardine	E		
	Metarhizium rileyi	NA	E (lepidopteran larvae)	*Quorum sensing*.	[7]
	Ophiocordyceps sinensis	-	E (larvae of *Thitarodes* spp.)	Insect hemolymph.	[8]
	Ophiocordyceps unilateralis	Zombie ant disease	E (ants)		[1]
Malasseziales	*Malassezia furfur*	Pityriasis (tinea versicolor)	H	L-DOPA.	
Mucorales	*Cokeromyces recurvatus*	Mucormycosis	M (occasionally, humans)	Temp., nutrients, O_2 & CO_2 tension.	[9]
	Mucor circinelloides		H (immunocompromised people)	O_2 tension.	[10]
	Mucor racemosus			O_2 & CO_2 tension.	[1, 11]
	Mucor rouxii				
Onygenales	*Histoplasma farciminosum*	Epizootic lymphangitis	M (mainly equids, but also humans, dogs, cats and badgers)	Temp.	[12]
	Paracoccidioides ceti		M (dolphins)		[13]
Ophiostomatales	*Ophiostoma novo-ulmi*	Dutch elm disease	P (*Ulmus procera*)	Quorum sensing and modifications in N_2 source.	[1, 3]
	Ophiostoma ulmi		P	N_2 source, *quorum-sensing*, linoleic acid.	
Sacharomycetales	*Candida albicans*	Candidiasis	H	Temp., N_2 starvation, blood glucose, serum, CO_2, pH, farnesol, GlcNAc.	[3, 11, 14]
	Holleya sinecauda	Mustard seed rot	P (mustard seeds)	Media solidity.	[1-3]
	Saccharomyces cerevisiae	-	Saprobe	C and N_2 starvation.	[3]
	Yarrowia lipolytica	Catheter-associated fungemia	H (rare opportunistic fungal pathogen; used in biotechnology)	C and N_2 source, serum, pH, temp., oxygenation, strain specificity.	[15]
Schizosaccharomycetales	*Schizosaccharomyces pombe*	-	Saprobe	N_2 starvation.	[3]
Taphrinales	*Taphrina deformans*	Leaf curl disease	P (peach and almond trees)	Unknown cue from host leaves.	[1-3]
Tremellales	*Cryptococcus neoformans*	Cryptococcosis	H	N_2 starvation.	[3]
Ustilaginales	*Sporisorium scitamineum*	Sugarcane smut	P (sugarcane)	Ammonium (?).	[16, 17]
	Ustilago maydis	Corn smut	P (maize and teosinte)	Pheromones, lipids, hydrophobicity, pH, N_2.	[1-3, 18]
Insertae sedis	*Tilletiopsis washingtonensis*	White haze	P (dead and living leaves, and post-harvest apples)	C source.	[3, 19]

Abbreviations: Temp. = temperature; E = entomopathogen; H = human pathogen; M = mammal pathogen; P= phytopathogen; Z = zoopathogen; (?) = doubtful trigger.

Table S2. Genes involved in virulence and or immune-evasion strategies in different TDF.

Taxa	Gene	Protein	Functions	Reference
Blastomyces dermatitidis	*BAD1*	*Blastomyces* adhesin-1 (bad-1)	Calcium binding protein involved in adhesion, phagocytosis, and modulation of host response.	[20]
	CDC11	Septin	Required for normal yeast and hyphae morphology (septins serve as a scaffold for proteins involved with cytokinesis, cell polarity, and cell morphology).	[21]
	CDC3			[22]
	CDC10			
	CDC12			
	DRK1	Dimorphism regulating histidine kinase (Drk1)	Dimorphic switching (senses host signals and triggers transition), osmotic stress adaptation, cell wall integrity regulation, and pathogenicity (it regulates the expression of other virulence genes).	[23]
	SREB	GATA transcription factor Sreb	Affects phase transition and regulates the biosynthesis of siderophores (iron-gathering molecules).	[24]
Coccidioides immitis	*SOWgp*	Spherule outer wall glycoprotein (SOWgp82)	It functions as an adhesin (binds laminin and fibronectin).	[25]
Coccidioides posadasii	*RYP1*	Required for yeast-phase transcription factor 1 (Ryp1)	Formation of mature spherules and colonization of host lungs (regulates morphology and virulence under host conditions, and genes involved in hyphal growth).	[26]
Emergomyces spp.	*CBP1*	Calcium binding protein 1 (Cbp1)	Growth in Ca limiting conditions and pathogenesis (macrophage lysis).	[27]
Histoplasma capsulatum	*AGS1*	∝-(1,3)-glucan synthase	Biosynthesis of ∝-(1,3)-glucan in the cell wall (pathogenesis).	[28]
	AMY1	∝-(1,4)-amylase		[29]
	CATB	Catalases (CatB and CatP)	Extracellular catalase activities. They catalyze the decomposition of hydrogen peroxide (H_2O_2), into oxygen and water. They also protect against other host-derived reactive oxygen species (ROS), produced by neutrophils, and activated macrophages.	[30]
	CATP		Intracelular catalase activities (protection against H_2O_2 *in vitro* ROS produced by neutrophils and activated macrophages).	
	CBP1	Cbp1	Allows growth in Ca limiting conditions and induces pathogenesis.	[31]
	DRK1	Drk1	Dimorphic switching, osmotic stress adaptation, cell wall integrity, and pathogenicity.	[23]
	HCL1	3-Hydroxy-methylglutaryl coenzyme A lyase	Inhibits phagosome acidification, vital for the survival during growth within macrophages.	[32]
	RYP1	Ryp1	Transcriptional regulator of dimorphic switching from mold to yeast at 37 °C (required for the expression of the vast majority of yeast-specific genes).	[33]
	RYP2	Ryp2 and Ryp3	Transcription factors involved in the control of cell morphology and regulation of sporulation at room temperature (both are essential for viable spore production).	[34]
	RYP3			
	RYP4	Ryp4	Transcription factor with a $Zn(II)_2Cys6$ cluster domain, essential component of the temperature-responsive Ryp regulatory network. Regulation of genes involved in yeast-phase growth.	[35]
	SID1	L-ornithine-N^5-monooxygenase	Necessary for siderophore biosynthesis, optimal intracellular growth, and pathogenesis.	[36]
	SOD3	Superoxide dismutase 3 (Sod3)	Detoxification of ROS, thus enabling yeast survival within phagocytes.	[37]
	SRE1	GATA transcription factor (Sre1)	Critical role in the regulation of the expression of genes related to siderophore biosynthesis and iron transportation and usage.	[38]
	VMA1	*vma1* (subunit A of vacuolar ATPase)	Involved in iron homeostasis during growth on iron limiting media, hyphal growth at 28 °C and pathogenesis.	[39]
Paracoccidioides spp.	*PbAOX*	Alternative oxidase (*aox*)	Involved in the intracellular redox balancing during host-*P. brasiliensis* interaction.	[40]
	PbCATA	Catalase (CatA, CatB and CatP, respectively)	ROS homeostasis (PbCatP protects against exogenous ROS; PbCatA and PbCatB against endogenous ROS).	[41]
	PbCATB			

Only genes with known function have been included. Note that some genes are present in different TDF, which gives an idea of their importance for virulence.

Table S2. (Continued).

Paracoccidioides spp.	*PbCATP*	Catalase (CatA, CatB and CatP, respectively)	ROS homeostasis (PbCatP protects against exogenous ROS; PbCatA and PbCatB against endogenous ROS).	[41]
	PbCBP1	Calcium binding protein (Cbp1)	Growth in Ca limiting conditions and pathogenesis (causes lysis of infected macrophages).	[27]
	PbCCP	Cytochrome C peroxidase (Ccp)	Avoids cell damage caused by nitrosative and oxidative stress and promotes fungal survival within macrophages.	[42]
	PbCDC42	Cdc42p (rho GTPase)	Coordination of bud emergence and yeast growth, cell cycle progression, cytokinesis, actin cytoskeleton remodeling, pseudohyphal growth, vesicle dynamics, and mating.	[43]
	PbDRK1	Drk1	Cell wall modulation (resistance to different cell wall-disturbing agents).	[44]
	PbGP43	Cell-surface component, gp43	Inhibits phagocytic and fungicidal activity of macrophages, reduces NO3 levels, and modulates host cells apoptosis.	[45]
	PbHAD32	Hydrolase PbHad32	Initial attachment of infectious particles to lungs. It may also participate at different stages of the morphological conversion.	[46]
	PbHSP90	Heat shock protein 90 (hsp90, ATP-dependent chaperone)	It regulates proliferation under ROS stress and controls cell differentiation (cooperates with calcineurin to control yeast to mycelium dimorphism) and promotes adaptation to host.	[47]
	PbP27	Protein p27	Important role in yeast cell architecture, glucose metabolism, and interaction with the monocyte/macrophage system. Possible role in promoting latency in the host.	[48, 49]
	Pb14-3-3	14-3-3 adhesin	Laminin binding (for attachment to host), morphological switching, ergosterol biosynthesis, and modulation of macrophages apoptosis.	[49]
	PbRBT5	rbt5	Putative hemoglobin receptor (may participate in iron acquisition pathway involved in hemoglobin iron uptake).	[50]
	PbSCONC	SconCp	Modulates inorganic sulfur metabolism (dimorphism regulator).	[51]
	PbSOD1	Cytosolic superoxide dismutase	Defense against endogenous-produced ROS.	[52]
	PbSOD3	Extracellular superoxide dismutase	Deletes superoxide radicals generated during host and pathogen interaction.	
Sporothrix schenckii	*SsDRK1*	Drk1	Global regulator of dimorphism and yeast-phase change (required for asexual development, yeast cell formation, cell wall composition and integrity, melanin synthesis, etc.).	[53]
	SsGp70	Gp70	Adhesin expressed on the fungal cell surface with key role in immunomodulation and host response (it binds to fibronectin, laminin, and type II collagen).	[54]
	SsCMK1	Calcium/calmodulin kinase I (sscmk1)	Morphological switching. It is involved in thermotolerance through interaction with Hsp90.	[55]
	SsHSP90	Hsp90	Response to heat shock and proteotoxic stress. It is needed for yeast cells growth at 35 °C.	
Talaromyces marneffei	*ABAA*	abaA	Controls of both conidiation and dimorphic switching.	[56]
	CFLA	Rho GTPase	Coordinately they control hyphal cell polarization. CflA regulates conidial germination and polarized growth of yeast. CflB controls polarized growth of conidiophores and hyphal branching.	[57]
	CFLB	Rho GTPase		
	CPEA	Catalase-peroxidase (cpeA)	Catalase-peroxidase (detoxification of H_2O_2).	[58]
	MP1	Mp1p	Immunogenic surface and secretory protein. Mediates virulence by improving survival within macrophages.	[59]

Table S3. LAMP assays for pathogenic and food/drink spoilage fungi (not necessarily dimorphic fungi).

Taxonomic order	Species name*	Molecular region	Sample origin**	Ref.
Chaetothyriales	*Cladophialophora carrionii*	*EF-1α*	H (chromoblastomycosis)	[60]
	Exophiala dermatitidis	*βTUB* (*BT2*)	H (phaeohyphomycosis)	[61]
Diaporthales	*Diaporthe phaseolorum*[1]	*EF1a*	P (soy beans)	[62]
Eurotiales	*Aspergillus carbonarius*	*PKS*	P (grapes)	[63]
	A. caelatus	*acl1*	P (peanuts, Brazil nuts, coffee beans)	[64, 65]
	A. flavus			
	A. fumigatus	*anxC4*	H (invasive aspergillosis)	[66]
		MAT	H (IA)	[67]
		cyp51A	H (IA), P (bulbs)	[68]
	A. nidulans	*hyd*	H (chronic granulomatous disease)	[69]
	A. niger	*PKS*	P (grapes)	[63]
	A. nomius	*amy1*	P (peanuts, Brazil nuts, coffee beans)	[64, 65]
	A. parasiticus			
	A. quadrilineatus	*hyd*	H (CGD)	[69]
	Penicillium expansum	*EX2044840*	P (fruits; blue mold decay)	[70]
Helotiales	*Oculimacula acuformis*	*βTUB*	P (cereal eyespot)	[71]
	O. yallundae			
Hypocreales	*Fusarium acuminatum*	*EF-1α*	P (medicinal herbs; root rot)	[72]
	F. cerealis	*Hyd5*	P (barley, malt; cereal head blight)	[73, 74]
	F. culmorum			
	F. graminearum			
		gaoA		[75, 76]
	F. solani	*EF-1α*	P (medicinal herbs; root rot)	[72]
Microascales	*Scedosporium apiospermum*[2]	*βTUB*	H (subcutaneous infections), O (environmental)	[77]
	S. aurantiacum			
	S. boydii[3]			
	S. dehoogii			
	S. prolificans			
	Pseudallescheria minutispora			
Mucorales	*Mucor racemosus*	*LSU* (*D1/D2*)	O (environmental)	[78]
Onygenales	*Trichophyton interdigitale*	*rDNA*	H (onychomycosis)	[79]
	T. rubrum			
Peronosporales	*Pythium insidiosum*	*rDNA*	H (pythiosis)	[80]
	Phytophthora kernoviae	*ITS*	P (crops; *Phytophthora* disease)	[81]
	P. lateralis			
	P. nicotianae			
	P. ramorum			
Pleosporales	*Leptosphaeria biglobosa 'brassicae'*	*ITS*	P (rapeseed; blackleg)	[82]
	L. biglobosa 'canadensis'			[82, 83]
	Pyrenochaeta lycopersici		P (crops; corky root)	
Pneumocystidales	*Pneumocystis jirovecii*[4]	*COX*	H (respiratory samples)	[85]
		COX (*cox2*)		[86]
		β-D glucan		[87]
		SSU		[88]
				[89]
	P. pneumonia	*SSU*		[90]
Saccharomycetales	*Candida albicans*	*ITS*	O (dairy product, yogurt)/ H (oral cytology samples)	[91, 92]
		LSU (*D1/D2*)	O (environmental)	[78]
	C. glabrata	*ITS*	O (dairy product, yogurt)	[91]

Species are ordered first by taxonomic order and then alphabetically. Abbreviations: *Acl1* = ATP citrate lyase subunit 1; *amy1* = alpha-amylase 1; *anxC4* = annexin C4; *β-D glucan* = (1→3)-β-D-glucan; *βTUB* = β-tubulin; *CAP* = capsule-associated gene; *CHS-1* = chitin synthase 1; *COX* = mitochondrial cytochrome c oxidase; *cyp51A* = cyp51A gene promoter region; *EF-1α* = elongation factor 1 alpha; *gaoA* = galactose oxidase; *Hyd* = hydrophobin; *IGS1* = intergenic spacer 1; *ITS* = internal transcribed spacer; *LSU* = nuclear large ribosomal subunit; *MAT* = mating type gene; *mtSSU* = mitochondrial small ribosomal subunit; *PKS* = calmodulin or polyketide synthase; rDNA = ribosomal operon (*SSU-ITS1-5.8S-ITS2-LSU*); *SSU* = nuclear small ribosomal subunit. *Former species name: [0]*Exophiala jeanselmei*; [1]*Phomopsis phaseoli*; [2]*Pseudallescheria boydii*; [3]*Pseudallescheria boydii* and *Petriellidium boydii*; [4]*Pneumocystis carinii*; ** Sample origin: A = animal; H = human (clinical); P = plant; O = other origin (food or drink); If applicable, either the name of the disease caused by the particular fungus, and/or the specific animal or plant affected appears between brackets.

Table S3. (Continued).

Saccharomycetales	*Candida parapsilosis*	*ITS*	O (dairy product, yogurt)	[91]
	C. tropicalis			
	Dekkera anomala		O (distilled water, wine, beer)	[93]
	D. bruxellensis			
	D. custersiana			
Sordariales	*Madurella mycetomatis*	*βTUB, ITS*	H (human mycetoma)	[94]
Tremellales	*Cryptococcus gattii*	*CAP59*	H (cryptococcal meningitis)	[95]
		CAP10		[96]
	C. neoformans	*CAP59*		[95]
		CAP10		[96]
		LSU (*D1/D2*)	O (environmental)	[78]
Trichosporonales	*Trichosporon asahii*	*IGS1*	O (dairy product, yogurt)/<br>H (invasive trichosporonosis)	[91, 97]
	T. mucoides		O (dairy product, yogurt)	[91]
Venturiales	*Ochroconis gallopava*	*LSU* (*D1/D2*)	H, A (cat, chicken, parakeet, turkey)	[98]

Table S4. Summary of LAMP and RCA assays designed so far for the diagnosis of endemic mycoses.

Target/<br>assay<br>(disease)	Taxa used for DNA extraction<br>(No. isolates)	No. samples<br>(infected<br>+ healthy patients)	ST/<br>SPC	Taxa used for specificity tests<br>(No. isolates)	Ref.
Hcp100/<br>LAMP<br>(HP)	*H. c.* strain H66 (1)<br>*H. c.* strain H68 (1)<br>*H. c.* strain H81 (2)<br>*H. c.* strain LAm B (5)<br>*H. c.* strain Netherland (1)<br>*H. capsulatum* var. *duboisii* (1)<br>*H. mississipiense* (4)<br>*H. ohiense* (65)<br>*H. suramericanum* (11)	Urine<br>(6 + 10)	67%/<br>100%	*Apophysomyces elegans* (2)<br>*Aspergillus* spp. (9)<br>***B. dermatitidis*** (2)<br>*Candida* spp. (7)<br>***Coccidioides immitis*** (1)<br>***C. posadasii*** (1)<br>*Cryptococcus* spp. (4)<br>*Fusarium* spp. (6)<br>*Lichtheimia corymbifera* (2)<br>*Microsporum equinum* (1)<br>*Mucor* spp. (2)<br>*Penicillium* spp. (2)<br>*Pneumocystis* spp. (3)<br>*Rhizopus oryzae* (2)<br>***Sporothrix schenckii*** (3)<br>*Trichophyton* spp. (3)	[99]
ITS/<br>LAMP<br>(HP)	*H. c.* strain H66 (1)<br>*H. c.* strain H69 (1)<br>*H. c.* strain H81 (1)<br>*H. c.* strain LAm B (1)<br>*H. c.* strain Netherland (1)<br>*H. capsulatum* var. *duboisii* (1)<br>*H. mississipiense* (1)<br>*H. ohiense* (1)<br>*H. suramericanum* (1)	Bone marrow (26*)<br>Whole blood<br>(1 + 5)<br>Heparinized blood spiked with yeasts (1)	54%/<br>95%	*Aspergillus* spp. (2)<br>*Candida* spp. (1)<br>*Cladophialophora carrionii* (1)<br>*Cryptococcus* spp. (2)<br>*Microsporum canis* (1)<br>***Paracoccidioides brasiliensis*** (1)<br>***Sporothrix brasiliensis*** (1)<br>*Trichosporon* spp. (2)	[100]
CAL/<br>RCA<br>(SP)	*S. brasiliensis* (25)<br>*S. schenckii* (58)<br>*S. globosa* (5)<br>*S. luriei* (1)<br>*S. mexicana* (4)<br>*S. pallida* (3)	Clinical samples (87)<br>Environmental, animal and plant samples (9)	NS/<br>100%	*Candida albicans* (1)<br>*Cryptococcus neoformans* (1)<br>*Histoplasma capsulatum* (1)<br>***Ophiostoma stenoceras*** (1)<br>*P. brasiliensis* (1)<br>***S. brunneoviolacea*** (1)<br>***S. chilensis*** (2)<br>***S. dimorphospora*** (1)<br>*Trichosporon asahii* (1)	[101]
ITS/<br>RCA<br>(HP)	*H. c.* strain LAm A (15)<br>*H. c.* strain LAm B (5)<br>*H. capsulatum* var. *duboisii* (1)<br>*H. farciminosum* (1)	Blood (1)<br>Bone marrow (8)<br>Bronchial lavage (1)<br>Lung (5)<br>Mucus (1)<br>Pustules or injuries (2)<br>Skin biopsy (1)<br>Other (6)<br>Unknown (11)	NS/<br>NS	*Aspergillus ochraceous* (1)<br>*Candida albicans* (1)<br>*Cryptococcus neoformans* (1)<br>*Leishmania braziliensis* (1)<br>***Paracoccidioides brasiliensis*** (1)<br>*Scedosporium* sp. (1)<br>*Trichophyton interdigitale* (1)	[102]

Taxa are ordered alphabetically. Those closely related to the genera *Histoplasma* and *Sporothrix* are in bold. Abbreviations: *H. c.* = *Histoplasma capsulatum*; HP = histoplasmosis; NS = not specified; SP = sporotrichosis; SPC = specificity; ST = sensitivity. *26 bone marrow samples were included, but only 11 corresponded to patients with positive cultures.

References in Tables S1–S4

1. Gauthier, G.M. Dimorphism in fungal pathogens of mammals, plants, and insects. *PLoS Pathog.* **2015**, *11*, e1004608.
2. Nadal, M.; García-Pedrajas, M.D.; Gold, S.E. Dimorphism in fungal plant pathogens. *FEMS Microbiol. Lett.* **2008**, *284*, 127–134.
3. Kijpornyongpan, T.; Aime, M.C. Investigating the smuts: Common cues, signaling pathways, and the role of MAT in dimorphic switching and pathogenesis. *JoF* **2020**, *6*.
4. McCorison, C.B.; Goodwin, S.B. The wheat pathogen *Zymoseptoria tritici* senses and responds to different wavelengths of light. *BMC Genomics* **2020**, *21*, 1–15.
5. Zhang, S.; Liang, M.; Naqvi, N.I.; Lin, C.; Qian, W.; Zhang, L.-H.; Deng, Y.Z. Phototrophy and starvation-based induction of autophagy upon removal of Gcn5-catalyzed acetylation of Atg7 in *Magnaporthe oryzae*. *Autophagy* **2017**, *13*, 1318–1330.
6. Rush, T.A.; Aime, M.C. The genus *Meira*: Phylogenetic placement and description of a new species. *Antonie Van Leeuwenhoek* **2013**, *103*, 1097–1106.
7. Boucias, D.; Liu, S.; Meagher, R.; Baniszewski, J. Fungal dimorphism in the entomopathogenic fungus *Metarhizium rileyi*: Detection of an in vivo quorum-sensing system. *J. Invertebr. Pathol.* **2016**, *136*, 100–108.
8. Liu, G.; Cao, L.; Qiu, X.; Han, R. *Quorum sensing* activity and hyphal growth by external stimuli in the entomopathogenic fungus *Ophiocordyceps sinensis*. *Insects* **2020**, *11*, 205.
9. Wondimu, B.; Bradley, B.; Lieberman, J.A.; Cohen, S.; Bui, L.; Reddi, D. *Cokeromyces recurvatus* Incidentally Found in a Patient with Gastric Outlet Obstruction. *Mycopathologia* **2022**, 1–6.
10. Valle-Maldonado, M.I.; Patiño-Medina, J.A.; Pérez-Arques, C.; Reyes-Mares, N.Y.; Jácome-Galarza, I.E.; Ortíz-Alvarado, R.; Vellanki, S.; Ramírez-Díaz, M.I.; Lee, S.C.; Garre, V.; et al. The heterotrimeric G-protein beta subunit Gpb1 controls hyphal growth under low oxygen conditions through the protein kinase A pathway and is essential for virulence in the fungus *Mucor circinelloides*. *Cell. Microbiol.* **2020**, *22*, e13236.
11. Pathan, E.K.; Ghormade, V.; Panmei, R.; Deshpande, M.V. Biochemical and molecular aspects of dimorphism in fungi. In *Advancing Frontiers in Mycology & Mycotechnology: Basic and Applied Aspects of Fungi*; T. Satyanarayana, S.K. Deshmu, and M.V. Deshpande, Eds.; Springer: Singapure, Republic of Singapure, 2019; pp. 69–94.
12. Jaleta, H.; Ameni, G.; Arage, M.; Giday; Girma, M.; Sori, T. *In vitro* evaluation of the effects of selected plants on the growth of the mycelial form of *Histoplasma capsulatum* variety *farciminosum* in Ethiopia. *J. Equine Vet. Sci.* **2020**, *91*, 103139.
13. Kanegae, H.; Sano, A.; Okubo-Murata, M.; Watanabe, A.; Tashiro, R.; Eto, T.; Ueda, K.; Hossain, M.A.; Itano, E.N. Seroprevalences against *Paracoccidioides cetii*: a causative agent for paracoccidiomycosis ceti (PCM-C) and *Coccidioides posadasii* for coccidioidomycosis (CCM) in Dall's porpoise (*Phocoenoides dalli*) and harbor porpoise (*Phocoena phocoena*) stranded at Hokkaido, Japan. *Mycopathologia* **2022**, *187*, 385–391.
14. Kinclová, O.; Potier, S.; Sychrová, H. The *Candida albicans* Na+/H+ antiporter exports potassium and rubidium. *FEBS Lett.* **2001**, *504*, 11–15.
15. Zieniuk, B.; Fabiszewska, A. *Yarrowia lipolytica*, a beneficious yeast in biotechnology as a rare opportunistic fungal pathogen: A minireview. *World J. Microbiol. Biotechnol.* **2018**, *35*, 10.
16. Bhuiyan, S.A.; Magarey, R.C.; McNeil, M.D.; Aitken, K.S. Sugarcane smut, caused by *Sporisorium scitamineum*, a major disease of sugarcane: a contemporary review. *Phytopathology* **2021**, *111*, 1905–1917.
17. Zhong, Y.; Yan, M.; Jiang, J.; Zhang, Z.; Huang, J.; Zhang, L.; Deng, Y.; Zhou, X.; He, F. Mycophenolic acid as a promising fungal dimorphism inhibitor to control sugar cane disease caused by *Sporisorium scitamineum*. *J. Agric. Food Chem.* **2018**, *67*, 112–119.
18. Gugnani, H.C. Dimorphism in plant and human fungal pathogens. *Kavaka, Trans. Mycol. Soc. India* **2022**, *58*, 43–48.

19. Baric, S.; Lindner, L.; Marschall, K.; Dalla Via, J. Haplotype diversity of *Tilletiopsis* spp. causing white haze in apple orchards in Northern Italy. *Plant Pathol.* **2010**, *59*, 535–541.
20. Brandhorst, T.T.; Wüthrich, M.; Warner, T.; Klein, B. Targeted gene disruption reveals an adhesin indispensable for pathogenicity of *Blastomyces dermatitidis*. *J. Exp. Med.* **1999**, *189*, 1207–1216.
21. Krajaejun, T.; Gauthier, G.; Rappleye, C.; Sullivan, T.; Klein, B. Development and application of a green fluorescent protein sentinel system for identification of RNA interference in *Blastomyces dermatitidis* illuminates the role of septin in morphogenesis and sporulation. *Eukaryot. Cell* **2007**, *6*, 1299–1309.
22. Marty, A.J.; Gauthier, G.M. *Blastomyces dermatitidis* septins CDC3, CDC10, and CDC12 impact the morphology of yeast and hyphae, but are not required for the phase transition. *Med. Mycol.* **2013**, *51*, 93–102.
23. Nemecek, J.C.; Wüthrich, M.; Klein, B.S. Global control of dimorphism and virulence in fungi. *Science* **2006**, *312*, 583–588.
24. Gauthier, G.M.; Sullivan, T.D.; Gallardo, S.S.; Brandhorst, T.T.; Vanden Wymelenberg, A.J.; Cuomo, C.A.; Suen, G.; Currie, C.R.; Klein, B.S. SREB, a GATA transcription factor that directs disparate fates in *Blastomyces dermatitidis* including morphogenesis and siderophore biosynthesis. *PLoS Pathog.* **2010**, *6*, e1000846.
25. Hung, C.-Y.; Yu, J.-J.; Seshan, K.R.; Reichard, U.; Cole, G.T. A parasitic phase-specific adhesin of *Coccidioides immitis* contributes to the virulence of this respiratory fungal pathogen. *Infect. Immun.* **2002**, *70*, 3443–3456.
26. Mandel, M.A.; Beyhan, S.; Voorhies, M.; Shubitz, L.F.; Galgiani, J.N.; Orbach, M.J.; Sil, A. The WOPR family protein Ryp1 is a key regulator of gene expression, development, and virulence in the thermally dimorphic fungal pathogen *Coccidioides posadasii*. *PLoS Pathog.* **2022**, *18*, e1009832.
27. Azimova, D.; Herrera, N.; Duvenage, L.; Voorhies, M.; Rodriguez, R.A.; English, B.C.; Hoving, J.C.; Rosenberg, O.; Sil, A. Cbp1, a fungal virulence factor under positive selection, forms an effector complex that drives macrophage lysis. *PLoS Pathog.* **2022**, *18*, e1010417.
28. Rappleye, C.A.; Engle, J.T.; Goldman, W.E. RNA interference in *Histoplasma capsulatum* demonstrates a role for α-(1,3)-glucan in virulence. *Mol. Microbiol.* **2004**, *53*, 153–165.
29. Marion, C.L.; Rappleye, C.A.; Engle, J.T.; Goldman, W.E. An α-(1, 4)-amylase is essential for α-(1, 3)-glucan production and virulence in *Histoplasma capsulatum*. *Mol. Microbiol.* **2006**, *62*, 970–983.
30. Holbrook, E.D.; Smolnycki, K.A.; Youseff, B.H.; Rappleye, C.A. Redundant catalases detoxify phagocyte reactive oxygen and facilitate *Histoplasma capsulatum* pathogenesis. *Infect. Immun.* **2013**, *81*, 2334–2346.
31. Sebghati, T.S.; Engle, J.T.; Goldman, W.E. Intracellular parasitism by *Histoplasma capsulatum*: fungal virulence and calcium dependence. *Science* **2000**, *290*, 1368–1372.
32. Isaac, D.T.; Coady, A.; Van Prooyen, N.; Sil, A. The 3-hydroxy-methylglutaryl coenzyme A lyase HCL1 is required for macrophage colonization by human fungal pathogen *Histoplasma capsulatum*. *Infect. Immun.* **2013**, *81*, 411–420.
33. Nguyen, V.Q.; Sil, A. Temperature-induced switch to the pathogenic yeast form of *Histoplasma capsulatum* requires Ryp1, a conserved transcriptional regulator. *PNAS* **2008**, *105*, 4880–4885.
34. Webster, R.H.; Sil, A. Conserved factors Ryp2 and Ryp3 control cell morphology and infectious spore formation in the fungal pathogen *Histoplasma capsulatum*. *PNAS* **2008**, *105*, 14573–14578.
35. Beyhan, S.; Gutierrez, M.; Voorhies, M.; Sil, A. A temperature-responsive network links cell shape and virulence traits in a primary fungal pathogen. *PLoS Biol.* **2013**, *11*, e1001614.
36. Hilty, J.; George Smulian, A.; Newman, S.L. *Histoplasma capsulatum* utilizes siderophores for intracellular iron acquisition in macrophages. *Med. Mycol.* **2011**, *49*, 633–642.

37. Youseff, B.H.; Holbrook, E.D.; Smolnycki, K.A.; Rappleye, C.A. Extracellular superoxide dismutase protects *Histoplasma* yeast cells from host-derived oxidative stress. *PLoS Pathog.* **2012**, *8*, e1002713.
38. Hwang, L.H.; Seth, E.; Gilmore, S.A.; Sil, A. SRE1 regulates iron-dependent and -independent pathways in the fungal pathogen *Histoplasma capsulatum*. *Eukaryot. Cell* **2012**, *11*, 16–25.
39. Hilty, J.; Smulian, A.G.; Newman, S.L. The *Histoplasma capsulatum* vacuolar ATPase is required for iron homeostasis, intracellular replication in macrophages and virulence in a murine model of histoplasmosis. *Mol. Microbiol.* **2008**, *70*, 127–139.
40. Hernández Ruiz, O.; Gonzalez, A.; Almeida, A.J.; Tamayo, D.; Garcia, A.M.; Restrepo, A.; McEwen, J.G. Alternative oxidase mediates pathogen resistance in *Paracoccidioides brasiliensis* infection. *PLoS Negl. Trop. Dis.* **2011**, *5*, e1353.
41. Tamayo, D.; Muñoz, J.F.; Almeida, A.J.; Puerta, J.D.; Restrepo, Á.; Cuomo, C.A.; McEwen, J.G.; Hernández, O. *Paracoccidioides* spp. catalases and their role in antioxidant defense against host defense responses. *Fungal Genet. Biol.* **2017**, *100*, 22–32.
42. Parente-Rocha, J.A.; Alves Parente, A.F.; Baeza, L.C.; Rondon Caixeta Bonfim, S.M.; Hernandez, O.; McEwen, J.G.; Bailão, A.M.; Pelleschi Taborda, C.; Borges, C.L.; de Almeida Soares, C.M. Macrophage interaction with *Paracoccidioides brasiliensis* yeast cells modulates fungal metabolism and generates a response to oxidative stress. *PLoS One* **2015**, *10*, e0137619.
43. Almeida, A.J.; Cunha, C.; Carmona, J.A.; Sampaio-Marques, B.; Carvalho, A.; Malavazi, I.; Steensma, H.Y.; Johnson, D.I.; Leão, C.; Logarinho, E.; et al. Cdc42p controls yeast-cell shape and virulence of *Paracoccidioides brasiliensis*. *Fungal Genet. Biol.* **2009**, *46*, 919–926.
44. Valente-Navarro, M.; Nascimento de Barros, Y.; Dias Segura, W.; Alencar Chaves, A.F.; Pereira Jannuzzi, G.; Spadari Ferreira, K.; Xander, P.; Luiz Batista, W. The role of dimorphism regulating histidine kinase (Drk1) in the pathogenic fungus *Paracoccidioides brasiliensis* cell wall. *JoF* **2021**, *7*, 1014.
45. Torres, I.; Hernandez, O.; Tamayo, D.; Muñoz, J.F.; Leitão, N.P., Jr.; García, A.M.; Restrepo, A.; Puccia, R.; McEwen, J.G. Inhibition of PbGP43 expression may suggest that gp43 is a virulence factor in *Paracoccidioides brasiliensis*. *PLoS One* **2013**, *8*, e68434.
46. Hernández, O.; Almeida, A.J.; Tamayo, D.; Torres, I.; Garcia, A.M.; López, A.; Restrepo, A.; McEwen, J.G. The hydrolase PbHAD32 participates in the adherence of *Paracoccidioides brasiliensis* conidia to epithelial lung cells. *Med. Mycol.* **2012**, *50*, 533–537.
47. Matos, T.G.; Morais, F.V.; Campos, C.B. Hsp90 regulates *Paracoccidioides brasiliensis* proliferation and ROS levels under thermal stress and cooperates with calcineurin to control yeast to mycelium dimorphism. *Med. Mycol.* **2013**, *51*, 413–421.
48. Torres, I.; Hernandez, O.; Tamayo, D.; Muñoz, J.F.; García, A.M.; Gómez, B.L.; Restrepo, A.; McEwen, J.G. *Paracoccidioides brasiliensis* PbP27 gene: knockdown procedures and functional characterization. *FEMS Yeast Res.* **2014**, *14*, 270–280.
49. da Silva, F.J.; Vicentim, J.; Oliveira, H.C.; Marcos, C.M.; Assato, P.A.; Andreotti, P.F.; Silva, J.L.; Soares, C.P.; Benard, G.; Almeida, A.M.; et al. Influence of the *Paracoccidioides brasiliensis* 14-3-3 and gp43 proteins on the induction of apoptosis in A549 epithelial cells. *Mem. Inst. Oswaldo Cruz* **2015**, *110*, 476–484.
50. Bailão, E.F.; Parente, J.A.; Pigosso, L.L.; de Castro, K.P.; Fonseca, F.L.; Silva-Bailão, M.G.; Báo, S.N.; Bailão, A.M.; Rodrigues, M.L.; Hernandez, O.; et al. Hemoglobin uptake by *Paracoccidioides* spp. is receptor-mediated. *PLoS Negl. Trop. Dis.* **2014**, *8*, e2856.
51. Menino, J.F.; Saraiva, M.; Gomes-Rezende, J.; Sturme, M.; Pedrosa, J.; Castro, A.G.; Ludovico, P.; Goldman, G.H.; Rodrigues, F. *P. brasiliensis* virulence is affected by SconC, the negative regulator of inorganic sulfur assimilation. *PLoS One* **2013**, *8*, e74725.
52. Tamayo, D.; Muñoz, J.F.; Lopez, Á.; Urán, M.; Herrera, J.; Borges, C.L.; Restrepo, Á.; Soares, C.M.; Taborda, C.P.; Almeida, A.J.; et al. Identification and analysis of the role of superoxide

dismutases isoforms in the pathogenesis of *Paracoccidioides* spp. *PLoS Negl. Trop. Dis.* **2016**, *10*, e0004481.
53. Zhang, Z.; Hou, B.; Wu, Y.Z.; Wang, Y.; Liu, X.; Han, S. Two-component histidine kinase DRK1 is required for pathogenesis in *Sporothrix schenckii*. *Mol. Med. Rep.* **2018**, *17*, 721–728.
54. Castro, R.A.; Kubitschek-Barreira, P.H.; Teixeira, P.A.; Sanches, G.F.; Teixeira, M.M.; Quintella, L.P.; Almeida, S.R.; Costa, R.O.; Camargo, Z.P.; Felipe, M.S. Differences in cell morphometry, cell wall topography and Gp70 expression correlate with the virulence of *Sporothrix brasiliensis* clinical isolates. *PLoS One* **2013**, *8*, e75656.
55. Rodriguez-Caban, J.; Gonzalez-Velazquez, W.; Perez-Sanchez, L.; Gonzalez-Mendez, R.; Valle, N.R.-d. Calcium/calmodulin kinase1 and its relation to thermotolerance and HSP90 in *Sporothrix schenckii*: an RNAi and yeast two-hybrid study. *BMC Microbiol.* **2011**, *11*, 1–16.
56. Borneman, A.R.; Hynes, M.J.; Andrianopoulos, A. The abaA homologue of *Penicillium marneffei* participates in two developmental programmes: conidiation and dimorphic growth. *Mol. Microbiol.* **2000**, *38*, 1034–1047.
57. Boyce, K.J.; Hynes, M.J.; Andrianopoulos, A. The Ras and Rho GTPases genetically interact to co-ordinately regulate cell polarity during development in *Penicillium marneffei*. *Mol. Microbiol.* **2005**, *55*, 1487–1501.
58. Pongpom, P.; Cooper, C.R., Jr; Vanittanakom, N. Isolation and characterization of a catalase-peroxidase gene from the pathogenic fungus, *Penicillium marneffei*. *Med. Mycol.* **2005**, *43*, 403–411.
59. Woo, P.C.Y.; Lau, S.K.P.; Lau, C.C.Y.; Tung, E.T.K.; Chong, K.T.K.; Yang, F.; Zhang, H.; Lo, R.K.C.; Cai, J.-P.; Au-Yeung, R.K.H.; et al. Mp1p is a virulence factor in *Talaromyces* (*Penicillium*) *marneffei*. *PLoS Negl. Trop. Dis.* **2016**, *10*, e0004907.
60. Deng, S.; de Hoog, G.S.; Pan, W.; Chen, M.; van den Ende, A.G.; Yang, L.; Sun, J.; Najafzadeh, M.J.; Liao, W.; Li, R. Three isothermal amplification techniques for rapid identification of *Cladophialophora carrionii*, an agent of human chromoblastomycosis. *J. Clin. Microbiol.* **2014**, *52*, 3531–3535.
61. Chen, M.; Kondori, N.; Deng, S.; Gerrits van den Ende, A.H.G.; Lackner, M.; Liao, W.; de Hoog, G.S. Direct detection of *Exophiala a*nd *Scedosporium* species in sputa of patients with cystic fibrosis. *Med. Mycol.* **2017**, *56*, 695–702.
62. Shen, H.; Wu, C.; Yang, H.; Song, B.; Zhang, H.; Wang, Y.; Zheng, X. The tef1α-LAMP method for rapid detection of *Diaporthe phaseolorum* var. *caulivora*. *J. Nanjing Agricult. Univ.* **2015**, *38*, 255–260.
63. Storari, M.; von Rohr, R.; Pertot, I.; Gessler, C.; Broggini, G.A.L. Identification of ochratoxin A producing *Aspergillus carbonarius* and *A. niger* clade isolated from grapes using the loop-mediated isothermal amplification (LAMP) reaction. *J. Appl. Microbiol.* **2013**, *114*, 1193–1200.
64. Luo, J.; Vogel, R.F.; Niessen, L. Development and application of a loop-mediated isothermal amplification assay for rapid identification of aflatoxigenic molds and their detection in food samples. *Int. J. Food Microbiol.* **2012**, *159*, 214–224.
65. Luo, J.; Taniwaki, M.H.; Iamanaka, B.T.; Vogel, R.F.; Niessen, L. Application of loop-mediated isothermal amplification assays for direct identification of pure cultures of *Aspergillus flavus*, *A. nomius*, and *A. caelatus* and for their rapid detection in shelled Brazil nuts. *Int. J. Food Microbiol.* **2014**, *172*, 5–12.
66. Jiang, L.; Gu, R.; Li, X.; Mu, D. Simple and rapid detection *Aspergillus fumigatus* by loop-mediated isothermal amplification coupled with lateral flow biosensor assay. *J. Appl. Microbiol.* **2021**, *131*, 2351–2360.
67. King, K.M.; Hawkins, N.J.; Atkins, S.; Dyer, P.S.; West, J.S.; Fraaije, B.A. First application of loop-mediated isothermal amplification (LAMP) assays for rapid identification of mating type in the heterothallic fungus *Aspergillus fumigatus*. *Mycoses* **2019**, *62*, 812–817.

68. Trabasso, P.; Matsuzawa, T.; Arai, T.; Hagiwara, D.; Mikami, Y.; Moretti, M.L.; Watanabe, A. Development and validation of LAMP primer sets for rapid identification of *Aspergillus fumigatus* carrying the cyp51A TR46 azole resistance gene. *Sci. Rep.* **2021**, *11*, 17087.
69. Matsuzawa, T.; Tanaka, R.; Horie, Y.; Gonoi, T.; Yaguchi, T. Development of rapid and specific molecular discrimination methods for pathogenic *Emericella* species. *Nippon Ishinkin Gakkai Zasshi* **2010**, *51*, 109–116.
70. Frisch, L.M.; Mann, M.A.; Marek, D.N.; Niessen, L. Development and optimization of a loop-mediated isothermal amplification (LAMP) assay for the species-specific detection of Penicillium expansum. *Food Microbiol.* **2021**, *95*, 103681.
71. King, K.M.; Eyres, G.J.; West, J.S.; Siraf, C.; Matusinsky, P.; Palicová, J.; Canning, G.G.M.; Bateman, G.L.; Fraaije, B.A.; Dyer, P.S. Novel multiplex and loop-mediated isothermal amplification assays for rapid species and mating-type identification of *Oculimacula acuformis* and *O. yallundae* (causal agents of cereal eyespot), and application for detection of ascospore dispersal and in planta use. *Phytopathology* **2021**, *111*, 582–592.
72. Wang, Y.; Wang, C.; Ma, Y.; Zhang, X.; Yang, H.; Li, G.; Li, X.; Wang, M.; Zhao, X.; Wang, J.; et al. Rapid and specific detection of *Fusarium acuminatum* and *Fusarium solani* associated with root rot on *Astragalus membranaceus* using loop-mediated isothermal amplification (LAMP). *Eur. J. Plant Pathol.* **2022**, *163*, 305–320.
73. Denschlag, C.; Vogel, R.F.; Niessen, L. Hyd5 gene based analysis of cereals and malt for gushing-inducing *Fusarium* spp. by real-time LAMP using fluorescence and turbidity measurements. *Int. J. Food Microbiol.* **2013**, *162*, 245–251.
74. Denschlag, C.; Vogel, R.F.; Niessen, L. Hyd5 gene-based detection of the major gushing-inducing *Fusarium* spp. in a loop-mediated isothermal amplification (LAMP) assay. *Int. J. Food Microbiol.* **2012**, *156*, 189–196.
75. Niessen, L.; Vogel, R.F. Detection of *Fusarium graminearum* DNA using a loop-mediated isothermal amplification (LAMP) assay. *Int. J. Food Microbiol.* **2010**, *140*, 183–191.
76. Abd-Elsalam, K.; Bahkali, A.; Moslem, M.; Amin, O.E.; Niessen, L. An optimized protocol for DNA extraction from wheat seeds and loop-mediated isothermal amplification (LAMP) to detect *Fusarium graminearum* contamination of wheat grain. *Int. J. Mol. Sci.* **2011**, *12*, 3459–3472.
77. Lu, Q.; Gerrits van den Ende, A.; Bakkers, J.; Sun, J.; Lackner, M.; Najafzadeh, M.; Melchers, W.; Li, R.; de Hoog, G. Identification of *Pseudallescheria* and *Scedosporium* species by three molecular methods. *J. Clin. Microbiol.* **2011**, *49*, 960–967.
78. Nakayama, T.; Yamazaki, T.; Yo, A.; Tone, K.; Alshahni, M.M.; Fujisaki, R.; Makimura, K. Detection of fungi from an indoor environment using loop-mediated isothermal amplification (LAMP) method. *Biocontrol Sci.* **2017**, *22*, 97–104.
79. Watanabe, S.; Okubo, A.; Miyajima, Y.; Satoh, K.; Makimura, K. Specific detection of *Trichophyton rubrum* and *Trichophyton interdigitale* based on loop-mediated isothermal amplification (LAMP) from onychomycosis specimens. *J. Dermatol.* **2019**, *46*, 1179–1183.
80. Htun, Z.M.; Rotchanapreeda, T.; Rujirawat, T.; Lohnoo, T.; Yingyong, W.; Kumsang, Y.; Sae-Chew, P.; Payattikul, P.; Yurayart, C.; Limsivilai, O.; et al. Loop-mediated Isothermal Amplification (LAMP) for Identification of *Pythium insidiosum*. *Int. J. Infect. Dis.* **2020**, *101*, 149–159.
81. Hieno, A.; Li, M.; Otsubo, K.; Suga, H.; Kageyama, K. Multiplex LAMP detection of the genus *Phytophthora* and four *Phytophthora* species *P. ramorum, P. lateralis, P. kernoviae,* and *P. nicotianae,* with a plant internal control. *Microbes Environ.* **2021**, *36*, ME21019.
82. Du, R.; Huang, Y.; Zhang, J.; Yang, L.; Wu, M.; Li, G.Q. LAMP detection and identification of the blackleg pathogen *Leptosphaeria biglobosa* '*brassicae*'. *Plant Dis.* **2021**, *105*, 3192–3200.
83. Du, R.; Luo, T.; Zhang, J.; Yang, L.; Wu, M.; Li, G. LAMP detection of the blackleg pathogen *Leptosphaeria biglobosa 'canadensis'*. *Crop Protect.* **2021**, *145*, 105610.

84. Hieno, A.; Naznin, H.A.; Suga, H.; Yamamoto, Y.Y.; Hyakumachi, M. Specific detection of Type 1 and Type 2 isolates of *Pyrenochaeta lycopersici* by loop-mediated isothermal amplification reaction. *Acta Agric. Scand., B Soil Plant Sci.* **2016**, *66*, 353–358.
85. Scharmann, U.; Kirchhoff, L.; Schmidt, D.; Buer, J.; Steinmann, J.; Rath, P.M. Evaluation of a commercial Loop-mediated Isothermal Amplification (LAMP) assay for rapid detection of *Pneumocystis jirovecii*. *Mycoses* **2020**, *63*, 1107–1114.
86. Huber, T.; Serr, A.; Geißdörfer, W.; Hess, C.; Lynker-Aßmus, C.; D. von Loewenich, F.; Bogdan, C.; Held, J. Evaluation of the amplex eazyplex loop-mediated isothermal amplification assay for rapid diagnosis of *Pneumocystis jirovecii* pneumonia. *J. Clin. Microbiol.* **2020**, *58*, e01739–20.
87. Nakashima, K.; Aoshima, M.; Ohkuni, Y.; Hoshino, E.; Hashimoto, K.; Otsuka, Y. Loop-mediated isothermal amplification method for diagnosing *Pneumocystis* pneumonia in HIV-uninfected immunocompromised patients with pulmonary infiltrates. *J. Infect. Chemother.* **2014**, *20*, 757–761.
88. Singh, P.; Singh, S.; Mirdha, B.R.; Guleria, R.; Agarwal, S.K.; Mohan, A. Evaluation of loop-mediated isothermal amplification assay for the detection of *Pneumocystis jirovecii* in immunocompromised patients. *Mol. Biol. Int.* **2015**, *2015*, 819091.
89. Zhang, H.; Liu, X.; He, R.; Jia, T.; Zhang, J. Establishment of loop-mediated isothermal amplification (LAMP) for detecting *Pneumocystis carinii*. *Chinese J. Parasitol. Parasitic Dis.* **2010**, *28*, 305–307.
90. Uemura, N.; Makimura, K.; Onozaki, M.; Otsuka, Y.; Shibuya, Y.; Yazaki, H.; Kikuchi, Y.; Abe, S.; Kudoh, S. Development of a loop-mediated isothermal amplification method for diagnosing *Pneumocystis pneumonia*. *J. Med. Microbiol.* **2008**, *57*, 50–57.
91. Kasahara, K.; Ishikawa, H.; Sato, S.; Shimakawa, Y.; Watanabe, K. Development of multiplex loop-mediated isothermal amplification assays to detect medically important yeasts in dairy products. *FEMS Microbiol. Lett.* **2014**, *357*, 208–216.
92. Noguchi, H.; Iwase, T.; Omagari, D.; Asano, M.; Nakamura, R.; Ueki, K.; Shinozuka, K.; Kaneko, T.; Tonogi, M.; Ohki, H. Rapid detection of *Candida albicans* in oral exfoliative cytology samples by loop-mediated isothermal amplification. *J. Oral Sci.* **2017**, *59*, 541–547.
93. Hayashi, N.; Arai, R.; Tada, S.; Taguchi, H.; Ogawa, Y. Detection and identification of *Brettanomyces/Dekkera* sp. yeasts with a loop-mediated isothermal amplification method. *Food Microbiol.* **2007**, *24*, 778–785.
94. Ahmed, S.A.; van de Sande, W.W.; Desnos-Ollivier, M.; Fahal, A.H.; Mhmoud, N.A.; de Hoog, G.S. Application of isothermal amplification techniques for identification of *Madurella mycetomatis*, the prevalent agent of human mycetoma. *J. Clin. Microbiol.* **2015**, *53*, 3280–3285.
95. Lucas, S.; da Luz Martins, M.; Flores, O.; Meyer, W.; Spencer-Martins, I.; Inácio, J. Differentiation of *Cryptococcus neoformans* varieties and *Cryptococcus gattii* using CAP59-based loop-mediated isothermal DNA amplification. *Clin. Microbiol. Infect.* **2010**, *16*, 711–714.
96. Tian, Y.; Zhang, T.; Guo, J.; Lu, H.; Yao, Y.; Chen, X.; Zhang, X.; Sui, G.; Guan, M. A LAMP-based microfluidic module for rapid detection of pathogen in cryptococcal meningitis. *Talanta* **2022**, *236*, 122827.
97. Zhou, J.; Liao, Y.; Li, H.; Lu, X.; Han, X.; Tian, Y.; Chen, S.; Yang, R. Development of a loop-mediated isothermal amplification assay for rapid detection of *Trichosporon asahii* in experimental and clinical samples. *BioMed Research International* **2015**, *2015*, 732573.
98. Ohori, A.; Endo, S.; Sano, A.; Yokoyama, K.; Yarita, K.; Yamaguchi, M.; Kamei, K.; Miyaji, M.; Nishimura, K. Rapid identification of *Ochroconis gallopava* by a loop-mediated isothermal amplification (LAMP) method. *Vet. Microbiol.* **2006**, *114*, 359–365.
99. Scheel, C.M.; Zhou, Y.; Theodoro, R.C.; Abrams, B.; Balajee, S.A.; Litvintseva, A.P. Development of a loop-mediated isothermal amplification method for detection of *Histoplasma capsulatum* DNA in clinical samples. *J. Clin. Microbiol.* **2014**, *52*, 483–488.

100. da Silva Zatti, M.; Domingos Arantes, T.; Lourenço Fernandes, J.A.; Baumgardt Bay, M.; Pipolo Milan, E.; Silva Naliato, G.F.; Cordeiro Theodoro, R. Loop-mediated isothermal amplification and nested PCR of the internal transcribed spacer (ITS) for *Histoplasma capsulatum* detection. *PLoS Negl. Trop. Dis.* **2019**, *13*, e0007692.
101. Rodrigues, A.M.; Najafzadeh, M.J.; de Hoog, G.S.; Pires de Camargo, Z. Rapid identification of emerging human-pathogenic *Sporothrix* species with rolling circle amplification. *Front. Microbiol.* **2015**, *6*, 1385.
102. Furuie, J.L.; Sun, J.; do Nascimento, M.M.F.; Gomes, R.R.; Waculicz-Andrade, C.E.; Sessegolo, G.C.; Rodrigues, A.M.; Galvão-Dias, M.A.; Pires de Camargo, Z.; Queiroz-Telles, F.; et al. Molecular identification of *Histoplasma capsulatum* using rolling circle amplification. *Mycoses* **2016**, *59*, 12–19.
